# The Metabolic Response of Skeletal Muscle to Endurance Exercise Is Modified by the ACE-I/D Gene Polymorphism and Training State

**DOI:** 10.3389/fphys.2017.00993

**Published:** 2017-12-14

**Authors:** Paola Valdivieso, David Vaughan, Endre Laczko, Michael Brogioli, Sarah Waldron, Jörn Rittweger, Martin Flück

**Affiliations:** ^1^Laboratory for Muscle Plasticity, Department of Orthopedics, Balgrist University Hospital, University of Zurich, Zurich, Switzerland; ^2^The Institute for Biomedical Research into Human Movement and Health, Manchester Metropolitan University, Manchester, United Kingdom; ^3^Functional Genomics Center Zurich, ETH, University of Zurich, Zurich, Switzerland; ^4^Institute of Aerospace Medicine, German Aerospace Center, Cologne, Germany; ^5^Department of Pediatrics and Adolescent Medicine, University of Cologne, Cologne, Germany

**Keywords:** muscle, exercise, endurance performance, muscle fibers, gene polymorphism, metabolism, metabolome analysis

## Abstract

The insertion/deletion polymorphism in the gene for the regulator of vascular tone, angiotensin-converting enzyme (ACE), is the prototype of a genetic influence on physical fitness and this involves an influence on capillary supply lines and dependent aerobic metabolism in skeletal muscle. The respective interaction of ACE-I/D genotype and training status on local metabolic and angiogenic reactions in exercised muscle is not known. Toward this end we characterized the metabolomic and angiogenic response in knee extensor muscle, *m. vastus lateralis*, in 18 untrained and 34 endurance-trained (physically active, $\dot{\text{V}}$O2max > 50 mL min^−1^ kg^−1^) white British men to an exhaustive bout of one-legged cycling exercise. We hypothesized that training status and ACE-I/D genotype affect supply-related muscle characteristics of exercise performance in correspondence to ACE expression and angiotensin 2 levels. ACE-I/D genotype and training status developed an interaction effect on the cross-sectional area (CSA) of *m. vastus lateralis* and mean CSA of slow type fibers, which correlated with peak power output (*r* ≥ 0.44). Genotype × training interactions in muscle also resolved for exercise-induced alterations of 22 metabolites, 8 lipids, glycogen concentration (*p* = 0.016), ACE transcript levels (*p* = 0.037), and by trend for the pro-angiogenic factor tenascin-C post exercise (*p* = 0.064). Capillary density (*p* = 0.001), capillary-to-fiber ratio (*p* = 0.010), systolic blood pressure (*p* = 0.014), and exercise-induced alterations in the pro-angiogenic protein VEGF (*p* = 0.043) depended on the ACE-I/D genotype alone. Our observations indicate that variability in aerobic performance in the studied subjects was in part reflected by an ACE-I/D-genotype-modulated metabolic phenotype of a major locomotor muscle. Repeated endurance exercise appeared to override this genetic influence in skeletal muscle by altering the ACE-related metabolic response and molecular aspects of the angiogenic response to endurance exercise.

## Introduction

Perfusion of the capillary bed is a critical determinant of maximal tissue metabolism as it sets the capacity for substrate uptake (Wagner, 1996; Bassett and Howley, 2000; Sarelius and Pohl, 2010). This dependence manifests with endurance training when most subjects demonstrate compensatory adaptations within the capillary bed of skeletal muscle, such as enhanced endothelium-dependent vasodilatation of arterioles and increased capillarisation (Egginton, 2009; Mortensen et al., 2014). The regulatory processes being implicated in exercise-induced vascular adaptations comprise a rapidly increased blood flow with the onset of muscle contractions (Clifford and Hellsten, 2004; Egginton, 2009). With the repetition of exercise the consequent elevation in microvascular shear stress stimulates in conjunction with abluminal mechanical factors morphogenic adaptations and proliferation of endothelial cells in muscle capillaries (Bassett and Howley, 2000; Gustafsson and Kraus, 2001; Prior et al., 2003). The consequent adaptations in muscle capillarisation manifest in a typical 20–40% increase in capillary density or capillary-to-fiber ratio after a sufficiently intense and long training stimulus such as exhaustive endurance exercise when sessions are repeated 5 times per week over 4–8 weeks (Hoppeler et al., 1985; Schmutz et al., 2010; Busso and Flück, 2013; Egan and Zierath, 2013). Thereby signs of capillary remodeling in exercised muscle are already detectable 3–12 h into recovery from a single exercise session by virtue of altered muscle expression of angiogenesis-associated gene transcripts (Clifford and Hellsten, 2004; Egginton, 2009; Schmutz et al., 2010).

Inhibition of vasoconstriction is a main factor contributing to the rapid increase in blood flow with muscle contraction (Clifford and Hellsten, 2004) and the subsequent angiogenic response (Mathes et al., 2015). The overriding of angiotensin 2-mediated constriction of peripheral blood vessels is a major regulatory switch for increased perfusion of muscle capillaries with exercise (Korthuis, 2011). Angiotensin 2 is produced through the action of endothelial-associated dipeptidipeptidyl-carbo-peptidase angiotensin converting enzyme (ACE) in perfused blood vessels and there is evidence for exercise-modulated production of angiotensin 2 production (Staessen et al., 1987; van Ginkel et al., 2015b). At rest, and in healthy subjects, angiotensin 2 production mainly arises from ACE being associated with the lung endothelium and kidney (Igic and Behnia, 2003; Brewster and Perazella, 2004). With intense exercise, angiotensin 2 levels in blood sizably increase (Staessen et al., 1987; van Ginkel et al., 2015a), pointing to the implication of ACE activity in organs which become increasingly perfused during exercise (Igic and Behnia, 2003; Maeda et al., 2005; Korthuis, 2011). Correlations of exercise-induced changes in serum angiotensin levels with capillary density (Vaughan et al., 2016) in skeletal muscle emphasize the possible contribution of a muscle-based ACE enzyme, aside the lung-based ACE activity, to angiotensin 2 production and angiotensin-regulated muscle perfusion with exercise (Vaughan et al., 2013, 2016; van Ginkel et al., 2015b).

The ACE-I/D gene polymorphism is a prominent sequence variation within the ACE gene, which exerts a distinct influence on the tissue expression and serum activity of ACE; and angiotensin 2 production (reviewed by Tiret et al., 1992; van Ginkel et al., 2015b; Vaughan et al., 2016). It is characterized by the insertion (I), or deletion (D), of a 287-basepair long silencer region in intron 16 of the ACE gene (Rigat et al., 1990); whereby the presence of the I-allele generally lowers stability of the ACE transcript (reviewed by Tiret et al., 1992; Mizuiri et al., 2001; Vaughan et al., 2013). ACE-I/D dependent vaso-regulation is reflected in a higher degree of capillary recruitment and perfusion with intense leg exercise in ACE-II genotypes that do not carry the D-allele (Vaughan et al., 2013, 2016; van Ginkel et al., 2015b). ACE-I/D genotype-related differences in the capacity for muscle perfusion develop a considerable effect on activity-induced metabolism. In young healthy subjects, this manifests through reduced aerobic combustion of glucose in mitochondria and up-regulated anaplerotic reactions in ACE-DD genotypes immediately after exhaustive endurance exercise on a cycle ergometer; giving rise to an exaggerated depletion of muscle glycogen with exhaustive endurance exercise (Vaughan et al., 2016). The ACE I/D genotype –dependent metabolic reactions are related to differences in capillarisation and mean cross sectional area of muscle fibers in a major knee extensor muscle, m. *vastus lateralis* (Vaughan et al., 2016). This metabolic differentiation supports an enhanced improvement in oxidative muscle characteristics, such as the volume density of subsarcolemmal mitochondria and intramyocellular lipid (Vaughan et al., 2013, 2016), in ACE- I-allele carriers with endurance training.

The mechanism underlying the effect of ACE involves a direct action of angiotensin 2 on the proliferation and differentiation of smooth muscle cells and endothelial cells, and indirect effects on endothelial cells through angiotensin 2 modulated shear stress which drives angiogenesis (Britten et al., 1999; Duncker and Bache, 2008; Clapp et al., 2009; Szostak and Laurant, 2011). In this regard we have recently reported that genetic and pharmacological inhibition of ACE influences the expression of pro-angiogenic and mitochondrial transcripts in leg muscle after bicycle-type endurance exercise (Mathes et al., 2015). Specifically, the expression of shear stress-related gene transcripts after exercise was enhanced, while expression of hypoxia-related transcripts was lowered under pharmacological inhibition of ACE (van Ginkel et al., 2016).

The ACE-I/D polymorphism has been calculated to account for only a fraction of inter-subject differences in ACE activity levels, serum angiotensin concentration and blood pressure (Danser et al., 2007). The findings indicate that additional factors, or confounders, which affect the ACE system and water homeostasis must play a role of regulation of ACE, such as prior exercise, thermal environment and water intake (Kosunen et al., 1976; Staessen et al., 1987; Danser et al., 2007). The endurance training state is possibly an important confounder, because it affects the capacity for exercise-induced muscle perfusion through an improvement in exercise-induced vasodilatation and increase of the capillary bed (Egginton et al., 1998; Flück and Hoppeler, 2003; Mortensen et al., 2014). In fact, endurance training state is an important factor, which affects the angiogenic response to exercise (Busso and Flück, 2013; Hoier et al., 2013), in association with improved vasodilatative and capacitive mechanism of muscle perfusion and aerobic metabolism (reviewed in Flück and Hoppeler, 2003; Clifford and Hellsten, 2004; Egginton, 2009; Korthuis, 2011; Mortensen et al., 2014). In turn this affects shear stress and tissue oxygenation-mediated control of angiogenic factor expression and action (Zumstein et al., 1983; Wibom et al., 1992; Egginton et al., 2001; Dapp et al., 2006; Williams et al., 2006). One aspect in this regard is the enhanced production and secretion of endothelium associated proteins during exercise as shown for VPF/VEGFA (vascular perfusion factor/vascular endothelial growth factor) (Hoier et al., 2013). Therefore, the aim of this investigation was to assess (1) whether the reported ACE-I/D genotype associated angiogenic and metabolic features of knee extensor muscle are modified by the endurance training state, and (2) whether the genetic effect is related to the local ACE system (ACE activity, transcript expression and angiotensin 2 blood serum concentration), the expression response of oxygenation- and shear stress-regulated pro-angiogenic proteins, VEGFA and tenascin-C, and metabolic reactions, which have been shown to depend on ACE-activity (Mathes et al., 2015; Vaughan et al., 2016), after exhaustive exercise. The latter aspect was addressed with an exploratory approach to identify muscle metabolite and lipid species that would demonstrate an influence of the ACE-I/D genotype and training status.

## Methods

### Subjects

Healthy, non-diabetic, 18–40 years old white men of British descent with a BMI between 20 and 30 kg m^−2^ were recruited via advertisement in local newspapers. In total, 52 subjects volunteered for the investigation. The study has been conducted with permission of the Ethics committee of Manchester Metropolitan University according to published guidelines (Harriss and Atkinson, 2011). All investigations were performed in accordance with the ethical standards of the 1964 Declaration of Helsinki. Written informed consent was obtained from every participant.

### Design

Physical activity levels, medical health, quality of life and dietary status of the subjects was assessed by a validated questionnaire (36-Item Short-Form Health Survey questionnaire; Ware and Sherbourne, 1992; Howley et al., 2003), basic anthropometry, imaging based assessment of body composition and quadriceps muscles. Aerobic fitness was estimated based on a two-legged $\dot{\text{V}}$O2peak test on a stationary cycle ergometer. One-week later subjects performed a standardized single bout of exhaustive one-legged cycle exercise under the collection of biopsies prior to, 0.5 and 8 h post cycling exercise from *vastus lateralis* muscle. The collected biopsies were used to quantify muscle composition, ACE transcript levels and activity, VEGFA and tenascin-C protein content, and muscle metabolites. The ACE-I/D polymorphism was determined in a double-blind manner from a mucosal swab as collected during the functional exploration and assessed post-hoc for its influence only after the physiological and biochemical measurements had been performed. For the analysis subjects were group ed based on their training status and ACE-I/D genotype as assessed by questionnaire, functional exploration and genotyping. Subjects were deemed to be endurance-trained if they documented a history of 5 years of regular endurance type training (endurance running, cycling, football, rugby), documented a level of intense physical activity above 6 h per week (in the 36-Item Short-Form Health Survey questionnaire), and if they demonstrated a $\dot{\text{V}}$O2peak above 50 mL min^−1^ kg^−1^. Healthy subjects which complied to the inclusion criteria, but which documented a level of intense physical activity below 6 h per week or demonstrated a $\dot{\text{V}}$O2peak below 50 mL min^−1^ kg^−1^ were considered as not being endurance-trained, i.e., being untrained.

### Imaging based assessment of body composition and quadriceps muscles

Whole body scans were run with Dual Energy X-ray Absorptiometry (DEXA) with a Lunar Prodigy Advance densitometer (GE Healthcare, Waukesha, Wisconsin, USA) to estimate body fat and calculate fat-free-mass as described previously (George et al., 1997). Participants lay supine on the scanning bed.

Magnetic resonance imaging (MRI) was used to measure cross-sectional area (CSA) within the quadriceps muscle group of the exercising leg (one-legged $\dot{\text{V}}$O2peak test). Participants were scanned using a 0.25-T G-scan MRI scanner (Esaote, Genova, Italy) in the supine position with the leg fully extended and relaxed, and held in place. A turbo 3D-T1-weighted protocol was used (matrix 256 × 256, TR 40 ms, TE 16 ms) and multiple 3.1-mm thick serial transverse sections were obtained every 25 mm from the distal to the proximal heads of the femur. Computing imaging software (OsiriX medical imaging software, OsiriX, Atlanta, USA) was used to determine the CSA of the *m. vastus lateralis* the quadriceps group at 50% of the femur length (Morse et al., 2007). The length of the femur was defined as the distance between the greater trochanter and the distal lateral condyle at the knee.

### Two-legged $\dot{\text{V}}$O2peak test

This assessment comprised ergospirometric measurements of aerobic performance, aerobic capacity and respiration during aerobic exercise tests to the limit of tolerance on an electrically braked cycle ergometer (Ergoline 800S; Ergoline GmbH, Bitz, Germany). Saddle length was adjusted to a position where the knee was extended at an ~175° angle when subjects were seated with the shoe heel placed on the pedal. The handlebar position was adjusted to a position allowing to a comfortable position. Power output, inspired and expired air (Cosmed K4b2, Italy), and heart rate was monitored with an Accurex Plus chest belt, Polar Electro Finland, Kempele, Finland).

The test started with a 4-min warm-up at 40 Watts at 80 rpm. Following the external work rate was increased by 5 Watts every 10 s until volitional exhaustion. The test was terminated when the pedal rate fell consistently below 70 rpm. Immediately following termination of the test, the participants were encouraged to perform an active recovery by cycling at a very low external workload. Test results were analyzed offline for absolute and specific, i.e., body mass-related peak oxygen uptake ($\dot{\text{V}}$O2peak), peak power output (PPO), and RER following exercise. $\dot{\text{V}}$O2peak was identified as the highest 30-s average oxygen uptake. RER at the beginning of the test was assessed from the average over a 15-s interval 2 min into the warm up. Maximum RER was determined from the average of a 15-s interval from the end of the test.

### Single bout of exhaustive one-legged cycle exercise

This stimulus was completed using the subject's dominant leg on the electrically braked cycle ergometer (Ergometrics Ergoline 800; Jaeger, Bitz, Germany) based on the values determined during the functional exploration.

Subjects were asked to reduce physical activity in the 3 days before the test and to report to the laboratory in the fasted state. The pedal on the side of the non-dominant leg was taken off and the corresponding non-active leg was placed on a fixed chair. Saddle length was set to the same value used for two-legged exercise. The exercising foot was then taped securely in place, with strong electrician's tape. Blood pressure was measured in a calm place in seated position with a sphygmomanometer after the subject has been sitting for at least 15 min. Subjects then performed the exercise bout to voluntary exhaustion. This started with a 5-min warm-up at a cadence of 80 rpm under and at 15% of the two-legged PPO. Next, the subjects exercised for 25 min at 30% of the two-legged PPO. Subsequently the intensity was ramped up in 10-Watt increments each minute until exhaustion. The intensity was not further increased when the pedal rate fell consistently below 60 rpm despite strong verbal encouragement. At the end of the exercise period, a 3-min cool-down phase was allowed at 15% of the calculated two-legged PPO.

Power output, inspired and expired oxygen and carbon dioxide was monitored during the exercise using a (Cosmed K4b2, Italy) and used to calculate absolute and relative peak power output and $\dot{\text{V}}$O2peak.

### Biopsy collection

Prior to exercise, a small (~50–100 mg) biopsy was taken under sterile conditions using a conchotome from the *vastus lateralis* muscle of the non-exercising leg. Biopsy position was standardized to 50% of femur length as assessed by prior ultrasound analysis. Lidocaine (2%) was infiltrated under the skin prior to performing the skin incision. The biopsy was placed on a wax plate, blotted dry and separated into two pieces. The first piece was frozen within 1 min after collection in liquid nitrogen cooled iso-pentane and stored in sealed 2-mL cryotubes (Nunc, Sigma, Buchs, Switzerland) at −80°C. The second sample was immediately mounted on cork (orientating the fibers longitudinally) and frozen within 3 min after collection using Tissue-Tek (Sakura, AJ Alphen aan den Rijn, The Netherlands), and then frozen in cold isopentane. All samples were then stored at −80°C until later analysis. 30-min post exercise fine needle biopsies were collected using a spring-loaded instrument (ACE-Onecut Disposable Biopsy, 14 Gauge × 150 mm, UK BIOPSY Ltd, Great Britain). Samples were frozen and stored as indicated above.

### Muscle composition

Mean CSA and percentage of muscle fiber types, capillary number and capillary-to-fiber ratio, were determined based on established protocols. In brief, cryo-sections were prepared from the frozen biopsy portion, which was mounted on cork. Care was taken to orient the cutting angle in perpendicular direction to the major axis of the muscle fibers.

For the assessment of muscle fiber types 10 μm-thick cryo-sections were quenched in 3% H_2_O_2_, blocked in 3% BSA/PBS and reacted in 0.3% BSA/PBS with slow (MAB1628, Chemicon-Merck Millipore, Burlington, Massachusetts, USA) or fast (My-32, Sigma Chemicals, Buchs, Switzerland) myosin-specific antibodies and horse radish peroxidise-conjugated anti-mouse IgG (A-2304 Sigma Chemicals, Buchs, Switzerland, 1:2,000) essentially as described previously (Vaughan et al., 2016). Immuno-reactivity was detected with substrate AEC (Sigma Chemicals, Buchs, Switzerland), the slides were mounted with Aquatex (MERCK) and non-overlapping microscopic fields of the stained section recorded using a light microscope (Carl Zeiss, Oberkochen, Germany). Fiber number and CSAs of type I (slow) and type II (fast) muscle fibers, respectively, were quantified using image J 1.6.0_33 J (http://imagej.nih.gov/ij) as described previously (Li et al., 2013). On average 130 fibers were measured per cross-section.

The number of capillaries per square millimeter (capillary density) and the capillary-to-fiber ratio was determined based on morphometric evaluation of lectin-stained, 14 μm-thick cryo-sections according to the published settings (van Ginkel et al., 2015a). On average 68 muscle fibers per cross-section were analyzed.

### Genotyping

The ACE-I/D genotype was determined essentially as described previously (Vaughan et al., 2016). The collected mucosal swab was frozen at −20°C in a sealed 15 mL tube (Sarstedt; Nümbrecht; Germany). DNA was extracted from the frozen swab after thawing with repeated vortexing the tip of the swap in 800 μL of methanol. The solution was air dried, frozen over-night at −80°C and resuspended in 100 μL of sterile water under heating to 65°C. DNA was recovered in the supernatant after a centrifugation step (5,000 g, 2 min, room temperature) and stored at −20°C. Genotyping for the ACE-I/D polymorphism with polymerase chain reaction (PCR) was carried out in a double-blind manner. Specifically, sample codes were blinded by a second investigator by sticking a label with random, but unique, four letter codes on top. The code was handed to a third investigator unrelated to the study. Subsequently the DNA samples were subjected together with mock and camouflage samples to a PCR reaction essentially as described previously (Evans et al., 1994). The primers corresponded to those established previously for the identification of the ACE-I/D genotype (for details see Genbank number X62855): detection of the 83-bp-amplicon specific to the absence of the insertion sequence (i.e., the D-allele) was achieved by a combination of ACE1 (5′-CATCCTTTCTCCCATTTCTC-3′) and ACE3 (5′-ATTTCAGAGCTGGAATAAAATT-3′) primers. ACE2 (5′-TGGGATTACAGGCGTGATACAG-3′) and ACE3 (5′-ATTTCAGAGCTGGAATAAAATT-3′) primers were applied to detect the 66-bp-amplicon specific for the I-allele in intron 16 of the ACE gene. PCR reactions were run with a mix of the three primers using Sybr Green master mix (Applied Biosystems) on an Applied Biosystems Real Time PCR system (SepOnePlus, Life Technologies). This involved 45 standard cycles of denaturing at 95°C for 15 s followed by annealing and extension at 55°C for 1 min. Amplicon identification followed using a melting curve analysis between a temperature range of 70°-80°C. The identity of the amplified sequences was validated by sequencing of the PCR product with specific primers (Microsynth, Balgach, Switzerland). Subsequently, the presence of the short amplicon for the I-allele was identified based on a lower melting temperature (73.5°C; 72.5–74°C) compared to the longer D-allele (75.5°C; 74.5–76.5°C) respectively. The genotyping results were decoded through the involvement of the third investigator once the functional test and metabolic measures had been completed.

### ACE transcript expression in skeletal muscle

RNA isolation and real-time polymerase chain reaction was carried out essentially as described previously (Zoll et al., 2006; van Ginkel et al., 2016). In brief, total RNA was isolated from a homogenate of pooled 20-μm thick cryo-sections corresponding to a volume of 10-mm3 biopsy using a rotor-stator homogenizer (Polytron PT1200, KINEMATICA AG, Lucerne, Switzerland) and the RNeasy Mini Kit (Qiagen, cat N° 74104) and Proteinase K (Qiagen, cat N° 19131). The isolated RNA was precipitated overnight at −20°C using 0.3 M sodium acetate (pH 5.2) in Ethanol and washed in 70% Ethanol. The dried RNA was resuspended in nuclease- free water (Fisher Scientific, Loughborough, United Kingdom) and purity and amount quantified using a spectrophotometer (at absorptions of 280, 260, and 240 nm). 100 ng RNA was reverse-transcribed using the OMNIscript Kit (Qiagen, cat N° 205110) with random hexamers, following the manufacturer's protocol. A volume corresponding to 3 or 0.3 ng (for 28S) initial RNA was subjected to real-time polymerase chain reaction (RT-PCR) with specific primers for ACE and 28S, using Sybr Green master mix (Applied Biosystems) on a Bio-Rad DNA machine (MJ/Bio-Rad Chromo4; Bio-Rad Laboratories, Watford, United Kingdom) controlled by the MJ Opticon Monitor software (Bio-Rad Laboratories, Watford, United Kingdom) based on the following protocol (50 standard cycles 15 s 95°C, 1 min 55°C, 15 s 75°C). The following primer sequences were used: ACE (5′-TCA CTA CGG GGC CCA GCA CA-3′; 5′-TGC GCC CAC ATG TTC CCC AG-3′). Primers were used according to established conditions (Zoll et al., 2006) and designed with the online NCBI Primer-Blast primer design tool (Rozen and Skaletsky, 2000) and synthesized at Sigma-Genosys. Amplification was assessed by a combination of analysis of melting curve and the cycle threshold. For amplification to be deemed successful there must have been a single clearly identifiable melting peak together with a clear exponential increase (sigmoid curve) and plateau. Transcript amounts were determined with the ΔCT method, taking individual efficiency of amplification into account. The calculated values were standardized to the input amount of cDNA, standardized to 28S and normalized to average values prior to exercise.

### ACE activity in skeletal muscle

The analysis of ACE activity in muscle tissue was carried out using a fluorometric assay as modified from the published method of Sentandreu and Toldra (2006). In brief, 10 mm3 of biopsy material from *vastus lateralis* muscle was cross-sectioned in a cryostat and homogenate was prepared in ice-cold 0.1 M KH2PO4 buffer using a Polytron®PT 1200E hand-held homogenizer (Kinematica AG, Lucerne, Switzerland). Protein concentration was determined using the BCA method (Pierce, Rockford IL, USA) against BSA as a standard.

Total homogenate corresponding to 35 μg protein in 100 μl 0.1 M KH2PO4 buffer was assessed by adding 200 μl of solution containing 0.45 mM of substrate o-aminobenzoylglycyl-p-nitro-L-phenylalanyl-L-proline (Abz-Gly-Phe(NO2)-Pro; cat. no. M-1100, Bachem, Bubendorf, Switzerland) and 1.125 M NaCl in 150 mM Tris-base buffer (pH 8.3). Reactions were run in duplicate for 4 h at 37°C in the dark in a flat bottom OptiPlate 96 well microplate (cat. No.6005290, Perkin Elmer, Perkin Elmer, Schwerzenbach, Switzerland). Fluorescence was measured using a Multi-detection Microplate Reader (SynergyTM HT, BioTek Instruments) at respective excitation and emission wavelengths of 360 and 460 nm. The coefficient of variation of repeated measures was 9%. The specificity of ACE-mediated substrate cleavage was monitored in control reactions where 1 μM Lisinopril (Ratiopharm, Madrid-Spain) was added. This was compared to signal of control incubations with 7.5 μg mL^−1^ of purified ACE enzyme (rabbit-lung, cat. no. A-6778; Sigma Buchs Switzerland) in the presence or absence of Lisinopril 1 μM. Under these conditions 96 and 75% of substrate conversion by the purified enzyme and total homogenate, respectively, was blocked with Lisinopril. The signal was calibrated vs. the emission of o-amino-benzoylglycine (Abz-Gly; Bachem cat. no. E-2920) as titrated between concentrations of 1.5 and 20 μM.

### Serum angiotensin 2 concentration

Blood (2 mL) was withdrawn from the venous cannula into vacutainers containing 60 μl Angiotensin 2 inhibitor cocktail, comprising 13.35 μl of O-Phenanthroline and Pepstatine A in DMSO mixed with 46.65 μl of EDTA and PHMB in aqueous solution (SPI bio, Bertin pharma, Versailles, France). The samples were immediately centrifuged at 10,000 rpm (3,000 g) and 4°C for 12 min. The supernatant was separated, snap frozen in liquid nitrogen and stored at −80°C until analyzed. Plasma was extracted with C18 phenyl cartridge, which were conditioned with 2 mL of methanol and then rinsed with 2 mL of water. Cold plasma (0.9 mL) was rapidly passed through the cartridge and subsequently washed with 1 mL of water. Absorbed angiotensins were eluted with 1 mL of methanol into conical polypropylene tubes. The eluate was evaporated to dryness by means of a nitrogen gas stream at room temperature and the residue was stored at −20°C. Angiotensin 2 was assessed with the angiotensin 2 enzyme immunoassay kit (SpiBio, Montigny Le Bretonneux, France). Briefly, the plasma samples were incubated for 1 h with 100 μl of EIA buffer (reconstituted the EIA buffer vial with 50 mL of distilled water), for 5 min with 50 μl of glutaraldehyde (100 μl of gluteraldehyde diluted in 0.125 mL of concentrated Wash buffer and 4.878 mL of distilled water), for 5 min with 100 μl of borane-trimethylamine (borane-trimethylamine vial diluted in 2.5 mL of 2N HCL and 2.5 mL of methanol) on a rocker platform, and with 100 μl of anti-angiotensin 2 IgG tracer (reconstituted the anti-antiotensin II-IgG tracer in 10 mL of EIA buffer) at 4° overnight. On the next day the samples were incubated with 200 μl of Ellman's reagent (Ellman's reagent vial in 1 mL of concentrated Wash buffer and 29 mL of distilled water). The plate was run with a single quick read on an Absorbance Microplate Reader (ELx800, BIO-TEK) with wavelength 405 after 30 min, 1 and 2 h of incubation.

After plotting the absorbance of each standard point vs. the concentration, the angiotensin 2 concentrations were calculated by interpolating from this standard curve. In order to reduce variability, angiotensin 2 values were normalized to the median of the pre-values.

### VEGF and tenascin-C protein expression in skeletal muscle

Total protein homogenate was prepared from the biopsy material, and subjected to SDS-PAGE and immunoblotting for tenascin-C and VEGF essentially as previously described (Flück et al., 2008; van Ginkel et al., 2016).

In brief, 5 mm3 of muscle tissue was extracted with a Polytron®PT 1200E hand-held homogenizer (Kinematica AG, Lucerne, Switzerland) from cross-sectioned 25-μm-thick cryo-sections into ice-cold RIPA buffer that included 10 mM Tris-HCl (pH 7.5), 150 mM NaCl, 1 mM EDTA, 1% NP-40, 2% Triton X100, 2 mM EDTA, 2 mM EGTA, one PhosStop tablet, and complete-mini EDTA-Free reagent (Roche Diagnostics GmbH, Mannheim, Germany). Protein concentration in the total homogenate was determined using the BCA method (Pierce, Rockford IL, USA) and adjusted to a concentration of 2 μg μL^−1^ using Laemmli buffer (Bio-Rad Laboratories AG, Cressier, Switzerland) including 2% mercaptoethanol. Samples were denatured for 5 min at 95°C and a volume corresponding to 10 μg of total protein from pre/post sample pairs was separated by 7.5% SDS-PAGE using precast gels (Bio-Rad Mini-protean TGX Stain-free). Proteins were blotted onto a nitrocellulose membrane with a Trans-blot-Turbo Transfer System (Bio-Rad AG, Cressier, Switzerland) and stained with Ponceau S to record protein loading based on the stained actin band at ~43 kDa. Subsequently the membrane was subjected to immunodetection with specific primary antibody against VEGFA (monoclonal antibody 26503; Abcam, Cambridge, United Kingdom) or tenascin-C (monoclonal antibody B28.13, a gift from Prof. Matthias Chiquet); and appropriate horse-radish peroxidase-conjugated secondary antibody in 5%-milk/10% BSA –TTBS (anti-mouse IgG raised in goat, A-2304, Sigma Buchs Switzerland). Signal was recorded with enhanced chemoluminescence (Supersignal West Femto, Fisher Scientific AG, Wohlen, Switzerland) using a PXi System (Syngene). Band signal intensity was estimated using the Quantity One software (Bio-Rad, Life Science Research, Hercules, CA, USA) with the “volume rectangular tool” and were corrected vs. the background of a band of equal height and size (area) from an empty sample lane. Background-corrected data were normalized to actin, and then normalized to the mean values of the pre-training sample from the same gel. Therefore, the final values reflect the relative expression levels per total muscle protein.

### Immunohistological detection of tenascin-C protein

Cryo-sections were prepared from *vastus lateralis* muscle, and subjected to immunological staining for VEGFA or tenascin-C using the MA3 antibody essentially as previously described (Flück et al., 2003). Nuclei were counterstained with hematoxylin.

### Muscle glycogen

Glycogen was measured relative to the total amount of muscle protein essentially as described previously (Vaughan et al., 2016). In brief, cryo-sections (25 μm) were prepared from muscle biopsies and the section volume estimated from microscopic measures of the CSA and the height of the sectioned tissue. An approximate of 1 mm^3^ tissue was homogenized in 100 μl of a PBS/inhibitor-cocktail [1 mL PBS + 9 mL dH_2_O + 1 complete Mini, EDTA-free tablet (Sigma Aldrich, Buchs, Switzerland) in a 1.5 mL Eppendorf tube by using a steel pistil (Behrens-Labortechnik, Germany). Total protein content was assessed using the Pierce BCA Protein Assay Kit (Thermo Scientific, Town, USA) and quantified at 562 nm on a 96-well plate with a Synergy HT spectrometer (BioTek Instruments Inc., Vermont USA). Glycogen was measured on 20 μl muscle homogenate against a glycogen standard with the Assay Kit (abcam, Cambridge, UK) according to the instructions. Signal was detected at 564 nm using a Synergy HT spectrometer (BioTek, Lucerne, Switzerland).

### Muscle metabolites

Metabolite profiles of biopsy samples, collected before and 30-min after one leg exercise were determined using ultrahigh performance liquid chromatography—tandem mass spectrometry (UPLC–MS) based on an established protocol (Vaughan et al., 2016). In brief, 5 mg muscle tissue was extracted in cold MetOH: MTBE: H20 = 360: 1,200: 348 using a full glass Potter type homogenizer. 10 μL of a 50-μM solution of each LysoPC (17:0) (Avanti Polar Lipids) and ^13^C-Sorbitol (Sigma) was added as internal standards. The non-polar and polar phase was separately recovered and stored at −30°C before being dried down under a steam of nitrogen and being reconstituted. For polar metabolites, this was 100 μL of 50 mM ammoniumacetate in acetonitrile – water 9:1 (v/v); for non-polar lipids, this was 100 uL of 10 mM ammonium acetate in 80% aqueous methanol. All extraction steps were carried out in dichlormethane-rinsed Duran glassware using MS-Grade compounds (CHROMOSOLV®, Sigma). All solvents used were of quality HPLC grade (Chromasolv, Sigma-Aldrich, Buchs, Switzerland).

Subsequently 1 μL of sample was injected (twice) at a flow of 3 μL min^−1^ into the UPLC-MS. Metabolites were separated on nanoAquity UPLC (Waters) equipped with a BEH-Amide capillary column (200 μm × 150 mm, 1.7 μm particle size, Waters), applying a gradient of 0.5 μM ammoniumacetate in acetonitril (A) and 0.5 μM ammoniumacetate in water (B) from 90 to 50% A. The UPLC was coupled to Q Exactive™ Hybrid Quadrupole-Orbitrap Mass Spectrometer (Thermo Fisher Scientific, Reinach, Switzerland) by a nanoESI source. MS data was acquired using negative polarization and all ion fragmentation (AIF) over a mass range of 80 to 1,200 m/z at a resolution of 70,000 (MS) and 25,000 (MSMS).

Lipid compounds were separated on nanoAquity UPLC (Waters) equipped with a C18 reversed phase column (HSS T3 1.7 microns, 0.2 × 50 mm, Waters) applying a gradient of 5 uM ammoniumacetate in acetonitril—water 40:60 v/v (A) and 5 uM ammoniumacetate in acetonitrile—isopropanol 10:90 v/v (B) from 95 to 2% A. The UPLC was coupled to a Q Exactive™ Hybrid Quadrupole-Orbitrap Mass Spectrometer (Thermo Fisher) by a nanoESI source that was operated in positive polarization mode, applying a voltage of 1.8 kV. The MS was operated in data independent acquisition mode (All Ion Fragmentation) with stepped collision energy setting (CE 30-35-40 V).

Mass spectrometry data sets were processed with Progenesis QI (Nonlinear Dynamics, A Waters Company) which aligns the ion intensity maps based on a reference data set, followed by a peak picking on an aggregated ion intensity map. Compound ions were detected and quantified by a multi-step procedure including the retention time (RT) alignment of observed m/z signals, the construction of a consensus m/z–RT map, grouping of co-eluting adducts with the major ions and quantification of detected compounds through all samples. Detected compound ions were further annotated by database searches based on derived neutral masses, isotopic patterns and the match of observed fragmentation spectra and theoretical fragmentation spectra. Database searches were run with a tolerance of 50 mD and included LipidMaps (www.lipidmaps.org) and the Human Metabolome Data Base (HMDB, www.hmdb.ca). Fragmentation patterns were not considered for the identifications. Compounds, which demonstrated significant differences (see paragraph “statistics” below), were allocated its respective HMDB or Pubchem substance identifier (SID).

Quality controls were run on individual and mixed samples to determine technical accuracy based on 20 selected compounds (amino acids, nucleotides, and metabolic intermediates) in mixed samples using Quan Browser (Xcalibur, Thermo Fisher Scientific) and 61 further abundant ions using Progenesis QI software (Nonlinear Dynamics). The coefficients of variation for biological and technical replicas of the 20 compounds in mixing experiments demonstrated values near or below 20%. Biological replicas correlated to an *r*-value of 0.72.

### Statistics

Data were organized in MS-Excel (Microsoft Office Professional Plus 10, Kildare, Ireland) and exported into SPSS 19.0 for statistical testing (version 23, IBM). Data were processed for display using Prism software (Graphpad Software Inc). Compliance with the Hardy-Weinberg equilibrium was assessed using an online calculator, i.e., https://www.cog-genomics.org/software/stats.

For performance, muscle composition and angiogenic parameters, effects of training status and genotype at rest and fold changes with exercise were calculated based on univariate ANOVAs for the factor training status (untrained, trained) and genotype. For the latter, the effect of the ACE_I/D genotype (ACE-DD, ACE-ID, ACE-II) or the effect of carrying the ACE I-allele [i.e., carriers (ACE-II or ACE-ID) vs. non-carriers (ACE-DD genotypes)] were assessed separately. The effect of exercise was assessed with a repeated ANOVA for the factor of exercise (pre, post). Fisher's *post-hoc* test was chosen to localize differences post hoc. Significance of a difference was declared at *p* < 0.05. Multiplicity error was controlled based on false discovery rate as proposed by Benjamini and Hochberg (Benjamini and Hochberg, 1995). Linear relationships were calculated based on Pearson correlations and called significant at *p* < 0.05. Association between training status and age were calculated based on Chi2-tests.

For the metabolomic analysis, data mining, subsequent analysis, and table assembly, was conducted in MS-Excel. Alterations in ion abundance were calculated only for those compounds that were detected in all analyzed samples. Signals were normalized to the summed abundance of all ions being detected in one given sample. For each sample, raw signals of each compound were related to the internal standard as generated by the Progenesis QI software. For multiply detected metabolite species, the average signal was calculated and used for the further analysis. Exercise induced changes were assessed based on permutations of *T*-tests using Significance Analysis of Microarrays test (SAM) running as an applet in MS-Excel software (Tusher et al., 2001) from a paired class comparison between the values from pre and 30-min post samples. A false discovery rate of 5% was deemed significant. Subsequently, compounds were identified which demonstrated an influence of the ACE-I allele in either training state (trained or untrained) based on *T*-tests. Affected metabolite and lipid compounds were subjected to supervised cluster analysis and displayed with Treeview as described previously (Valdivieso et al., 2017).

## Results

### Cell physiological characteristics of the subjects in relation to training state

**Figure 1:**
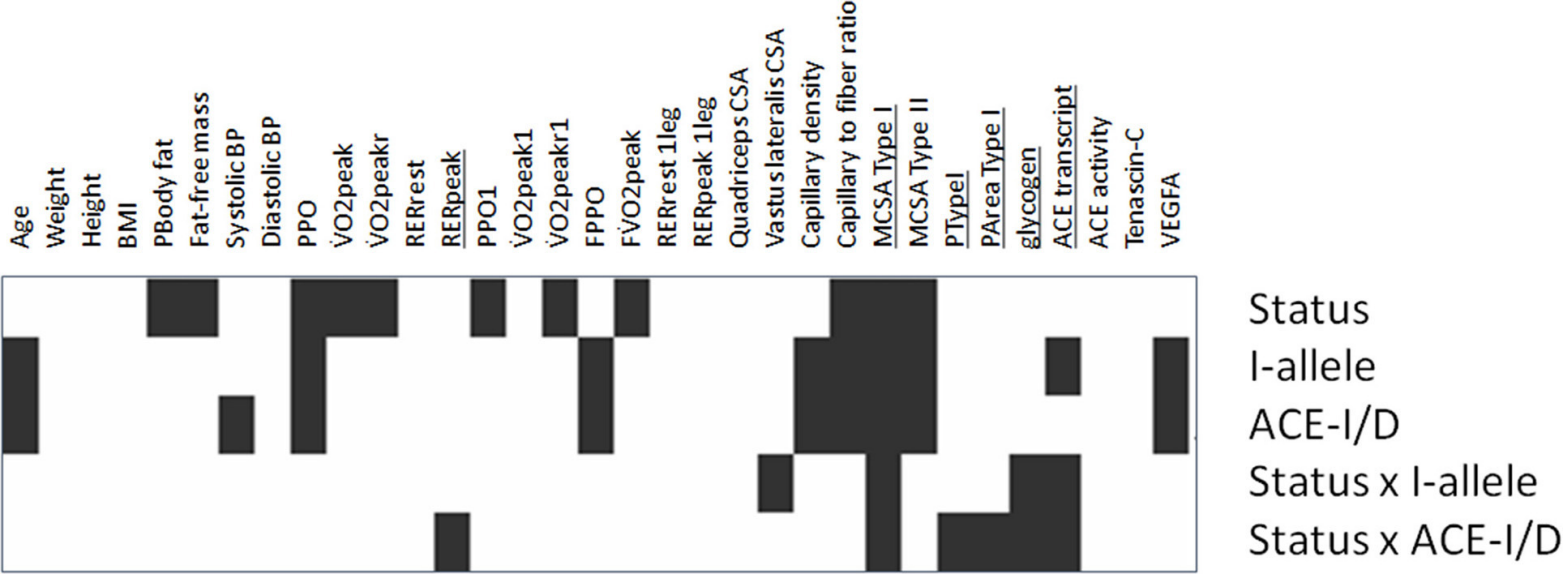
Significance map of training × genotype interactions for parameters of muscle-powered endurance exercise. Visualization of the *p*-values for the indicated effects for the anthropometric, physiological and muscle data of the studied subjects (untrained: 11xACE-DD, 10xACE-ID, 3xACE-II; trained: 15xACE-DD, 10xACE-ID, 3xACE-II) before exercise based on univariate ANOVAs. Black rectangles indicate those effects, which passed the 5% threshold of statistical significance Underlined font highlights parameters, which demonstrated an interaction effect between “training status” × “genotype.” PArea TypeI, percentage of the muscle biopsy CSA being composed of type I muscle fibers; PTypeI, percentage of type I muscle fibers.

Twenty-eight of the 52 studied subjects classified to the criteria of being endurance-trained; the other 24 subjects being classified as untrained. Table 1 summarizes the physiological characteristics of the subjects in dependence of the training status. Trained subjects had less body fat (14.2 vs. 22.2%) and more fat-free-mass (64.6 vs. 59.8 kg) than the untrained subjects (Table 1). Training status influenced performance related parameters of endurance performance (Figure 1). This comprised a 23.1% higher peak aerobic power (PPO), and 20.3% higher absolute peak oxygen uptake ($\dot{\text{V}}$O2peak), respectively. There was a trend for a larger CSA of the quadriceps group in the trained compared to untrained subjects (*p* = 0.051; 2319.9 vs. 2013.6 mm2; Table S2). MCSAs of type I (+36.2%) and type II (+27.2%) muscle fibers, and capillary-to-fiber ratio (+27.3%) in *m. vastus lateralis* were larger in the trained than the untrained subjects (Table S2).

**Table 1:** Subject characteristics.

	**untrained or trained (*****n*** = **52)**		**Untrained (*****n*** = **24)**		**trained (*****n*** = **28)**		***p*-value**
Age (years)	28.1 ± 0.9	(19.0–38.0)	26.3 ± 1.14	(19.0–37.4)	28.1 ± 1.2	(19.0–38.0)	0.2605
Mass (kg)	77.6 ± 1.6	(58.3–99.0)	77.7 ± 2.41	(59.7–99.0)	79.9 ± 2.4	(58.3–99.0)	0.5194
Height (cm)	180.3 ± 1.6	(155.7–204.4)	180.9 ± 2.72	(158.4–204.4)	181.7 ± 0.0	(1.6–2.0)	0.8391
BMI (kg m^−2^)	23.9 ± 0.5	(18.5–30.9)	24.0 ± 0.73	(18.5–30.9)	24.1 ± 0.7	(19.4–29.6)	0.9330
Body fat (%)	17.3 ± 1.1	(6.0–32.1)	22.2 ± 6.92	(10.7–32.1)	14.2 ± 1.3	(6.0–28.0)	0.0030
Fat-free- mass (kg)	64.6 ± 1.3	(48.5–79.6)	59.8 ± 1.6	(48.5–71.6)	64.6 ± 1.2	(58.7–79.6)	0.0004
Systolic BP (mm Hg)	123.8 ± 1.6	(104.9–148.0)	124.0 ± 2.27	(104.9–134.3)	125.3 ± 2.2	(113.1–148.0)	0.6785
Diastolic BP (mm Hg)	74.3 ± 1.3	(62.3–92.0)	74.0 ± 2.10	(63.7–83.5)	75.3 ± 2.0	(62.3–92.0)	0.6664
PPO (Watt)	315.8 ± 8.4	(229.5–467.5)	293.4 ± 11.35	(229.5–387.6)	361.1 ± 11.6	(252.0–467.5)	0.0001
$\dot{\text{V}}$O2peak (mL O2 min^−1^)	4107.5 ± 102.3	(2983.5–5816.8)	3815.7 ± 148.89	(2983.5–5102.9)	4589.3 ± 152.4	(3315.0–5816.8)	0.0008
$\dot{\text{V}}$O2peakr (mL O2 min^−1^ kg^−1^)	53.8 ± 1.2	(38.7–72.7)	48.8 ± 1.70	(38.7–61.4)	59.5 ± 1.7	(49.9–72.7)	0.0001
RERrest ($\dot{\text{V}}$CO2/$\dot{\text{V}}$ O2)	0.77 ± 0.01	(0.58–0.99)	0.77 ± 0.02	(0.58–0.99)	0.72 ± 0.03	(0.63–0.89)	0.2156
RERpeak ($\dot{\text{V}}$CO2/$\dot{\text{V}}$ O2)	1.11 ± 0.02	(0.84–1.32)	1.10 ± 0.03	(0.84–1.32)	1.07 ± 0.19	(0.97–1.30)	0.6640

*Values represent mean ± SE of values (minima-maxima) of the studied subjects combined (n = 52) and split for endurance training status (trained n = 28; untrained n = 24). P-values refer to the significance of differences between the trained and untrained group (univariate ANOVA for ACE-I/D allele × training status as a factor). Underlined p-values denote those differences, which passed the threshold of a false discovery rate of 5%. BMI, body mass index; BP, blood pressure; PPO, peak power output (during two-legged exercise); RERpeak, peak respiration exchange ratio during two-legged exercise; RERrest, respiration exchange ratio at rest before two-legged exercise; $\dot{\text{V}}$O2peak, peak oxygen uptake (during two-legged exercise); $\dot{\text{V}}$O2peakr, body mass-related peak oxygen uptake during two-legged exercise*.

### ACE-I/D genotype-associated exercise performance

Hardy-Weinberg equilibrium was met in the 52 studied subjects (i.e., *p* = 0.483). ACE-I/D genotype effects were seen for systolic blood pressure at rest, PPO, and FPPO (Figure 1, Table S1). I-allele carriers developed 11% more PPO than non-carriers (*p* = 0.014). At the post hoc level, PPO during two-legged exercise (*p* = 0.014), and PPO1 during one-legged exercise (*p* = 0.040), were both 19% higher in ACE-II than ACE-DD genotypes. In trained subjects PPO during two-legged exercise remained 14% higher in ACE-II than ACE-DD genotypes (Table S1). An interaction effect of the ACE-I/D genotype and training status was identified for RERpeak (Table S1).

### Training status and ACE-I/D genotype-associated muscle parameters

**Figure 2:**
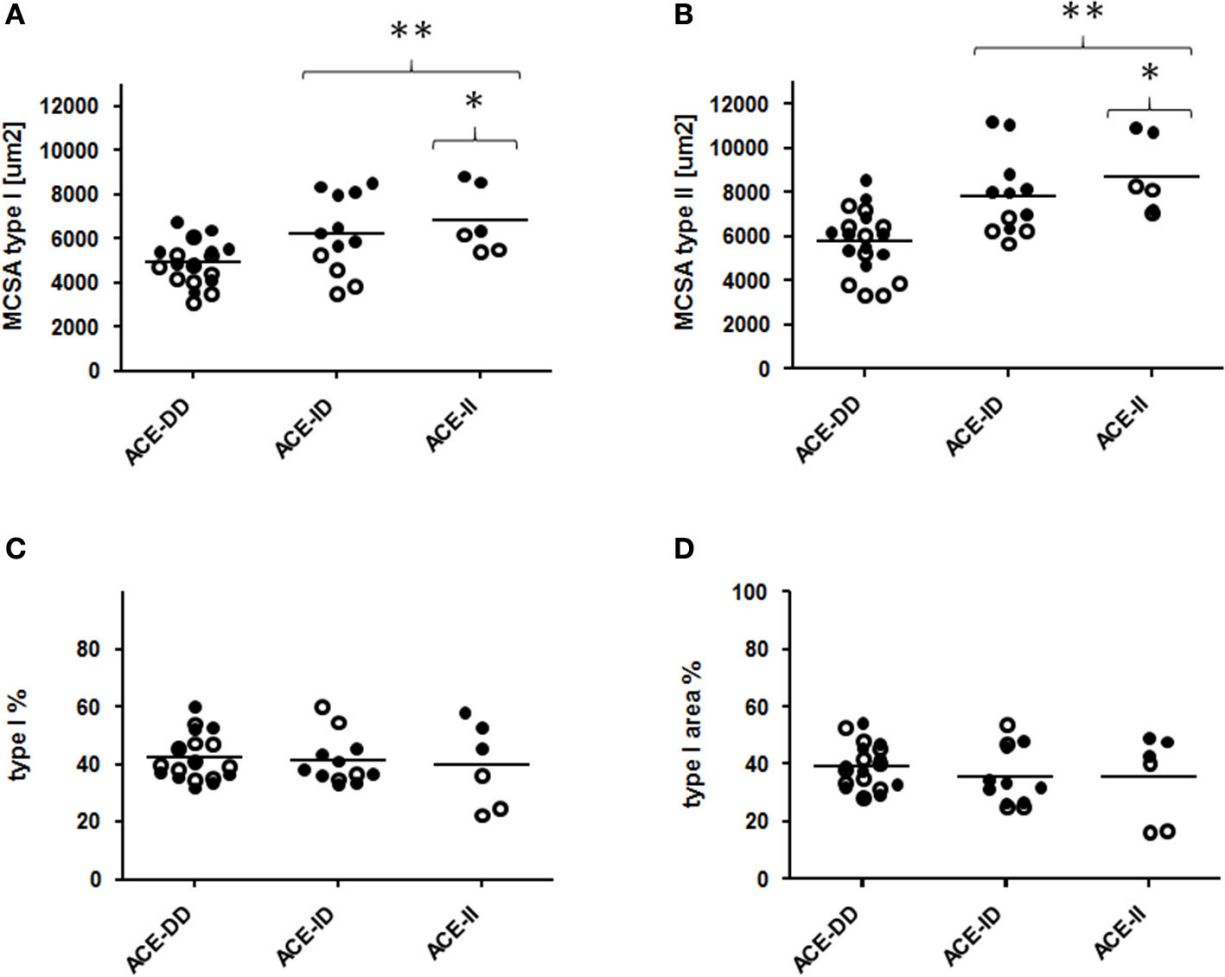
Effect of ACE-I/D genotype on muscle fiber structure. Scatter dot plot of the individual values and means (horizontal lines) of mean cross-sectional area (MCSA) of type I and II muscle fibers **(A,B)**, distribution **(C)** and area percentage **(D)** of type I muscle fibers for the 52 studied subjects when split for ACE-I/D genotype (26xACE-DD, 20xACE-ID, 6xACE-II). Filled and non-filled symbols in A, B indicate values of the trained and untrained subjects, respectively. ^*^ and ^**^ denote *p* < 0.05 and *p* < 0.01 vs. ACE-DD. Univariate ANOVA with post hoc test of Fisher. Only genotype effects are indicated.

**Figure 3:**
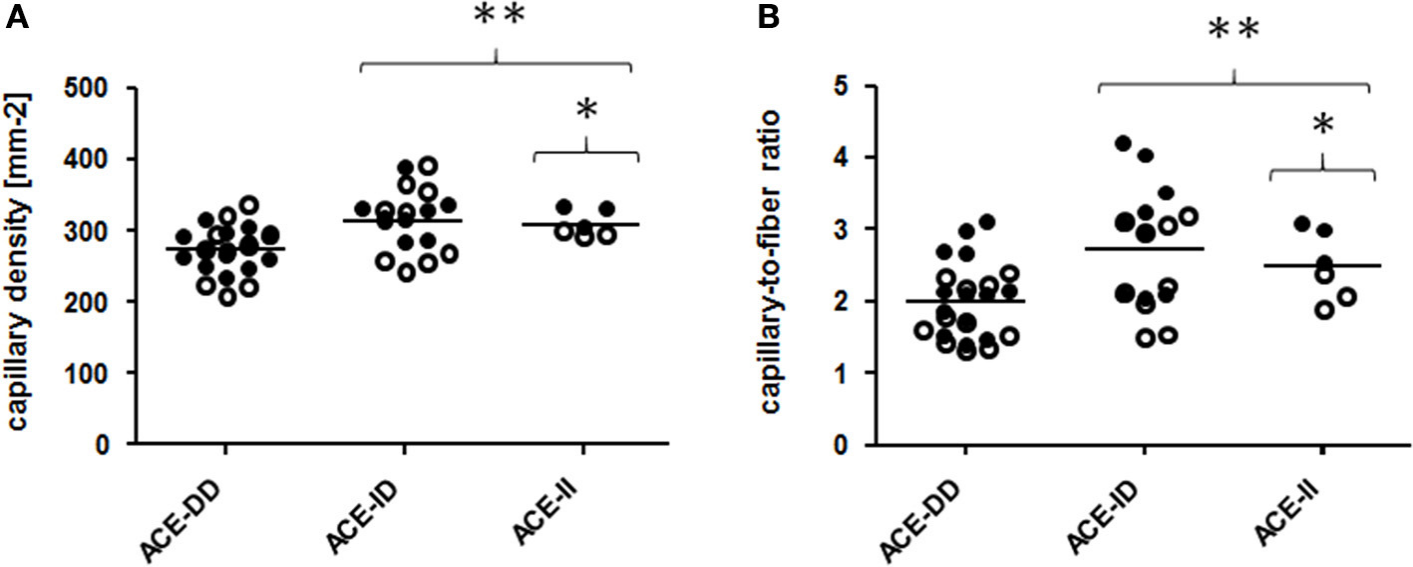
Effect of ACE-I/D genotype on muscle capillarisation. Scatter dot plot of the individual values and means of capillary density **(A)** and capillary to fiber ratio **(B)** for the 52 studied subjects when split for ACE-I/D genotype (26xACE-DD, 20xACE-ID, 6xACE-II). Filled and non-filled symbols in **(A,B)** indicate values of the trained and untrained subjects, respectively. ^*^ denotes ^*^p < 0.05 vs. ACE-DD; ^**^p < 0.01 vs. ACE-DD. Univariate ANOVA with post hoc test of Fisher. Only genotype effects are indicated.

Genotype effects were seen for the composition of *vastus lateralis* muscle, including capillary density and capillary-to-fiber ratio, MCSA of type I and II muscle fibers (Figure 1, Table S2). The muscle CSA and MCSA for type I muscle fibers, the percentage of type I fibers and the area percentage of type I muscle fibers in the middle portion of *m. vastus lateralis*, demonstrated an interaction effect between training state × ACE-I/D genotype (Table S2). Untrained carriers of the ACE I-allele had a 22.6% larger CSA than untrained non-carriers of the ACE I-allele (i.e., ACE-DD genotypes; *p* = 0.040). Trained I-allele carriers demonstrated a 38.8% larger MCSAs of type I muscle fibers than trained non-carriers of the I-allele (Figures 2A,B). Capillary-to-fiber ratio and capillary density were elevated in ACE I-allele compared to non-I-allele carriers of the ACE gene irrespective of the training state (Table S2, Figures 3A,B).

### One-legged exercise stimulus

Table 2 shows the physiological characteristics of the subjects during the one-legged exercise stimulus on the cycle ergometer in dependence of the training state. During the one-legged exercise, trained subjects performed at an 8% lower fraction of two-legged $\dot{\text{V}}$O2peak (F$\dot{\text{V}}$O2peak) than the untrained individuals. One-legged cycling exercise increased RER by 15% in the untrained subjects and 13% in the trained subjects, relative to pre-values.

**Table 2:** Physiological characteristics of trained and untrained subjects during one-legged exercise.

**Factor**	**untrained or trained (*n* = 52)**	**untrained (*n* = 24)**	**trained (*n* = 28)**	
	**Mean ±SD**	**Mean ±SD**	**Mean ±SD**	***p*-value**
PPO1 (W)	190.8 ± 41.6	177.8 ± 38.3	221.1 ± 43.7	0.0011
$\dot{\text{V}}$O2peak1 (mLO2 min^−1^)	3425.7 ± 630.8	3352.7 ± 672.3	3678.4 ± 765.6	0.1409
$\dot{\text{V}}$O2peakr1 (mLO2 min^−1^ kg^−1^)	43.5 ± 5.3	43.2 ± 5.4	45.8 ± 6.2	0.1478
FPPO (fraction)	0.60 ± 0.07	0.60 ± 0.06	0.61 ± 0.07	0.8667
F$\dot{\text{V}}$O2peak (fraction)	0.83 ± 0.09	0.88 ± 0.09	0.81 ± 0.10	0.0143
RERrest1 ($\dot{\text{V}}$CO2/$\dot{\text{V}}$O2)	0.81 ± 0.17	0.79 ± 0.13	0.82 ± 0.16	0.5755
RERpeak1 ($\dot{\text{V}}$CO2/$\dot{\text{V}}$O2)	0.92 ± 0.10	0.91 ± 0.09	0.93 ± 0.11	0.5792

*Values represent mean ± SD of baseline (i.e., pre) values of the studied subjects combined (n = 52) and split for endurance trained (n = 28) and untrained (n = 24) status. P-values refer to the significance of differences between the trained and untrained group (univariate ANOVA for ACE-I/D allele x training status as a factor) with values that passed a false discovery rate of 5% being underlined. PPO1, peak power output during one-legged exercise; FPPO, fraction of PPO at which the one-legged exercise was performed; F$\dot{\text{V}}$O2peak, fraction of $\dot{\text{V}}$O2peak at which the one-legged exercise was performed; RERpeak1, peak respiration exchange ratio during one-legged exercise; RERrest1, respiration exchange ratio before the one-legged exercise; $\dot{\text{V}}$O2peak1, peak oxygen uptake during one-legged exercise; $\dot{\text{V}}$O2peakr1, body mass-related peak oxygen uptake during one-legged exercise*.

### Training state influences the ACE-I/D genotype modulated angiogenic response to exercise

**Figure 4:**
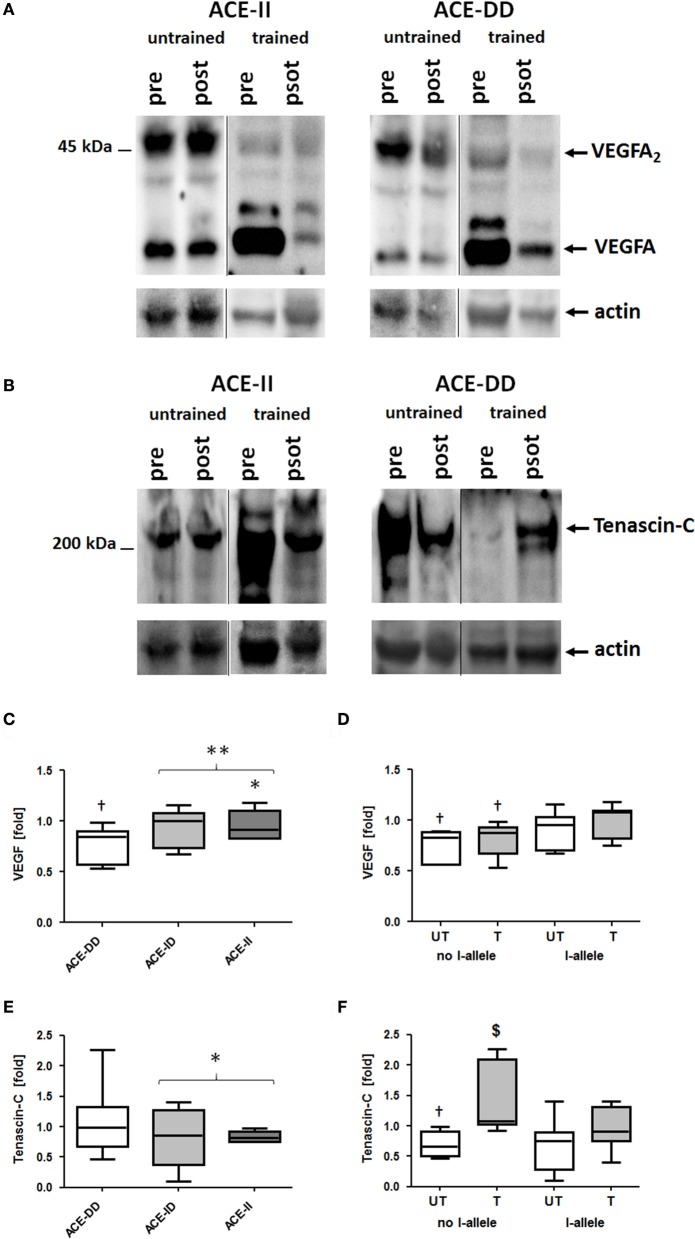
Effect of ACE-I/D genotype and training status on pro-angiogenic protein levels in vastus lateralis muscle. Examples of the detection of tenascin-C and VEGFA expression in *vastus lateralis* muscle pre- and post-exercise in homogenates of untrained and trained ACE-DD and ACE-II genotypes **(A,B)**. Actin loading controls are shown below. **C–F**) Box-and-whisker plot of median fold changes in VEGFA **(C,D)** and Tenascin-C **(E,F)** 30-min post exercise vs. pre- for the 52 studied subjects when split for ACE-I/D genotype (**C,E**; 26xACE-DD, 20xACE-ID, 6xACE-II) or training state and the presence of the ACE I-allele (**D**, **F**; untrained: untrained: 11xACE-DD, 10xACE-ID, 3xACE-II; trained: 15xACE-DD, 10xACE-ID, 3xACE-II). The lines denote the median, the box delimits the quartiles and the whiskers denote the farthest points that are not outliers. †*p* < 0.05 vs. pre; ^*$*^*p* < 0.05 vs. same genotype untrained; ^*^ and ^**^ denote *p* < 0.05 and *p* < 0.01 vs. ACE-DD (no I-allele), respectively. Univariate ANOVA with post hoc test of Fisher. Only genotype effects are indicated.

VEGFA protein was detected as a monomer and dimer at 23 and 46-kDa, respectively (Figure 4). Tenascin-C protein was detected mainly as the 230-kDa isoform (Figure 4).

At rest, prior to exercise VEGFA protein levels in *m. vastus lateralis* were higher in carriers of the ACE I-allele than non-carriers, irrespective of whether subjects were untrained (+92%) or trained (+90%; Table S2). VEGFA protein levels were 13% reduced 30-min after one-legged exercise and this response was affected by the ACE I-allele (*p* = 0.003), but not the training status (*p* = 0.181; Figures 4C,D). The effect was explained by a reduction in VEGFA protein levels in non-carriers of the ACE I-allele compared to the I-allele carriers (−22 vs. −6%; Figure 4D).

Tenascin-C protein levels at rest were not affected by training status (*p* = 0.243) and ACE-I/D genotype (*p* = 0.846) and I-allele (*p* = 0.536) prior to exercise. Exercise-induced alterations in tenascin-C levels demonstrated an effect of training status (*p* = 0.002) and trend for effect of the ACE-I-allele (*p* = 0.050) and trend for an interaction effect of training status × ACE I-allele (*p* = 0.064; Figure 4F). In untrained non-carriers of the I-allele (i.e., ACE-DD) tenascin-C protein levels were 30% decreased post exercise, whereas trained ACE-DD genotypes demonstrated 47% elevated tenascin-C levels post exercise.

### ACE-I/D genotype modifies the local ACE system

Prior to one-legged exercise, serum angiotensin 2 levels were 238% higher, in non-carriers than carriers of the ACE I-allele (*p* = 0.003, data not shown). ACE transcript levels in *vastus lateralis* muscle prior to exercise demonstrated an interaction effect between ACE-I/D genotype and training status (Table S2). Untrained non-carriers of the ACE I-allele showed 38% higher ACE transcript levels than the respective I-allele carriers whereas trained non-carriers of the ACE I-allele showed 70% lower ACE transcript levels than I-allele carriers. ACE activity in muscle tissue prior to exercise did not differ dependent on ACE-I/D genotype (*p* = 0.569), the I-allele (*p* = 0.929) and training status (*p* = 0.286).

**Figure 5:**
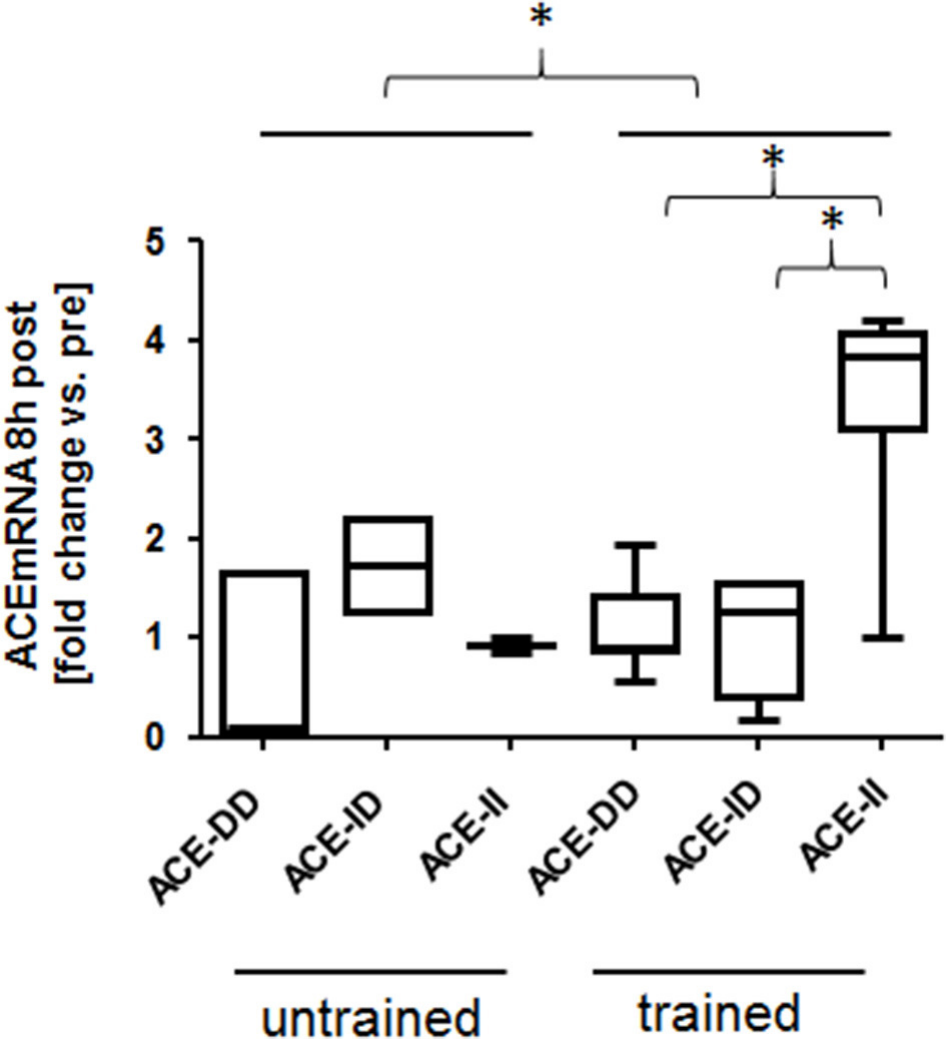
Effect of ACE-I/D genotype and training status on ACE transcript expression post exercise. Box-and-whisker plot of fold alterations in ACE transcript levels between the trained and untrained subjects, 8 h post- vs. pre-exercise, in each ACE-I/D genotype. ^*^p < 0.05 for the indicated comparison. ACE-II. Univariate ANOVA with post hoc test of Fisher.

One-legged exercise affected ACE transcript expression 8-h post exercise. Exercise-induced fold changes in ACE transcript levels demonstrated an effect of training state (*p* = 0.031), ACE-I/D genotype (0.017) and an interaction effect between genotype × training status (*p* = 0.003; Figure 5). The effect was explained by a selective increased ACE transcript levels in trained homozygous I allele carriers (i.e., ACE-II genotypes; Figure 5).

### Training state influences the ACE-I/D genotype modulated metabolic response to exhaustive exercise

**Figure 6:**
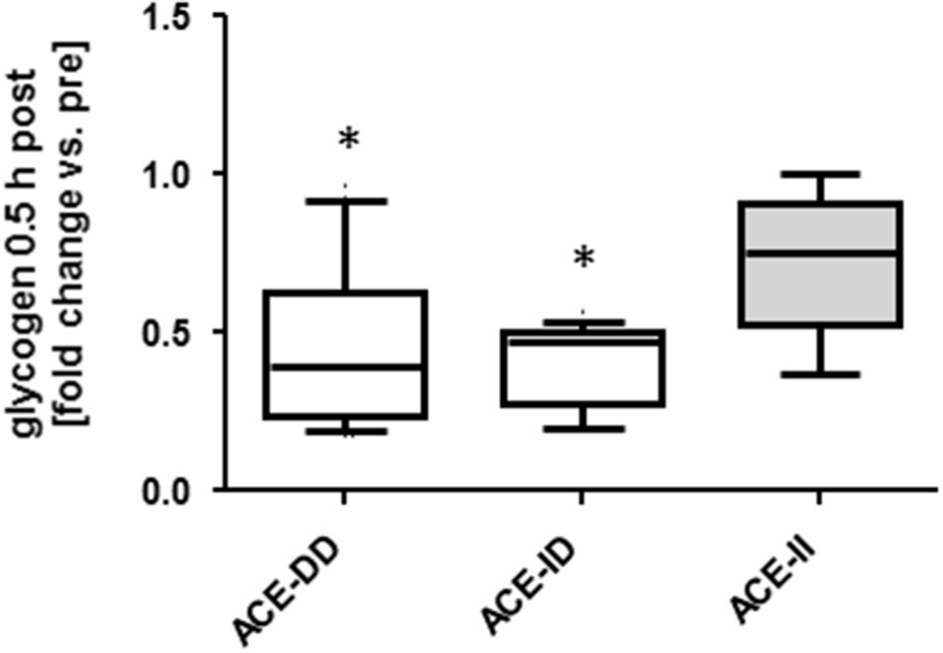
Effect of ACE-I/D genotype on glycogen depletion post exercise. Box-and-whisker plot of fold alterations in glycogen concentration in the studied subjects, 30-min post vs. pre-exercise, in each ACE-I/D genotype. ^*^p < 0.05 vs. ACE-II. Univariate ANOVA with post hoc test of Fisher.

We explored the acute metabolic effects of one-legged exercise in a subset of subjects (i.e., 8 untrained and 14 endurance-trained subject). At rest before exercise, glycogen concentration demonstrated an ACE-I/D genotype × training status interaction (Table S2), i.e., for the untrained subjects being 0.05 mg/mg higher in the ACE-DD than ACE-II genotypes, while for the trained subjects being 0.06 mg/mg lower in ACE-DD than the ACE-II genotypes. Glycogen concentration was reduced 30-min after the one-legged exercise in both untrained and trained subjects. The exercise-induced fold changes in glycogen concentration depended on the ACE-I/D genotype (*p* = 0.010) but not the training status (*p* = 0.677). Post exercise glycogen concentration was 42% and 36% more reduced in ACE-ID and ACE-DD than ACE-II genotypes, respectively (Figure 6).

**Figure 7:**
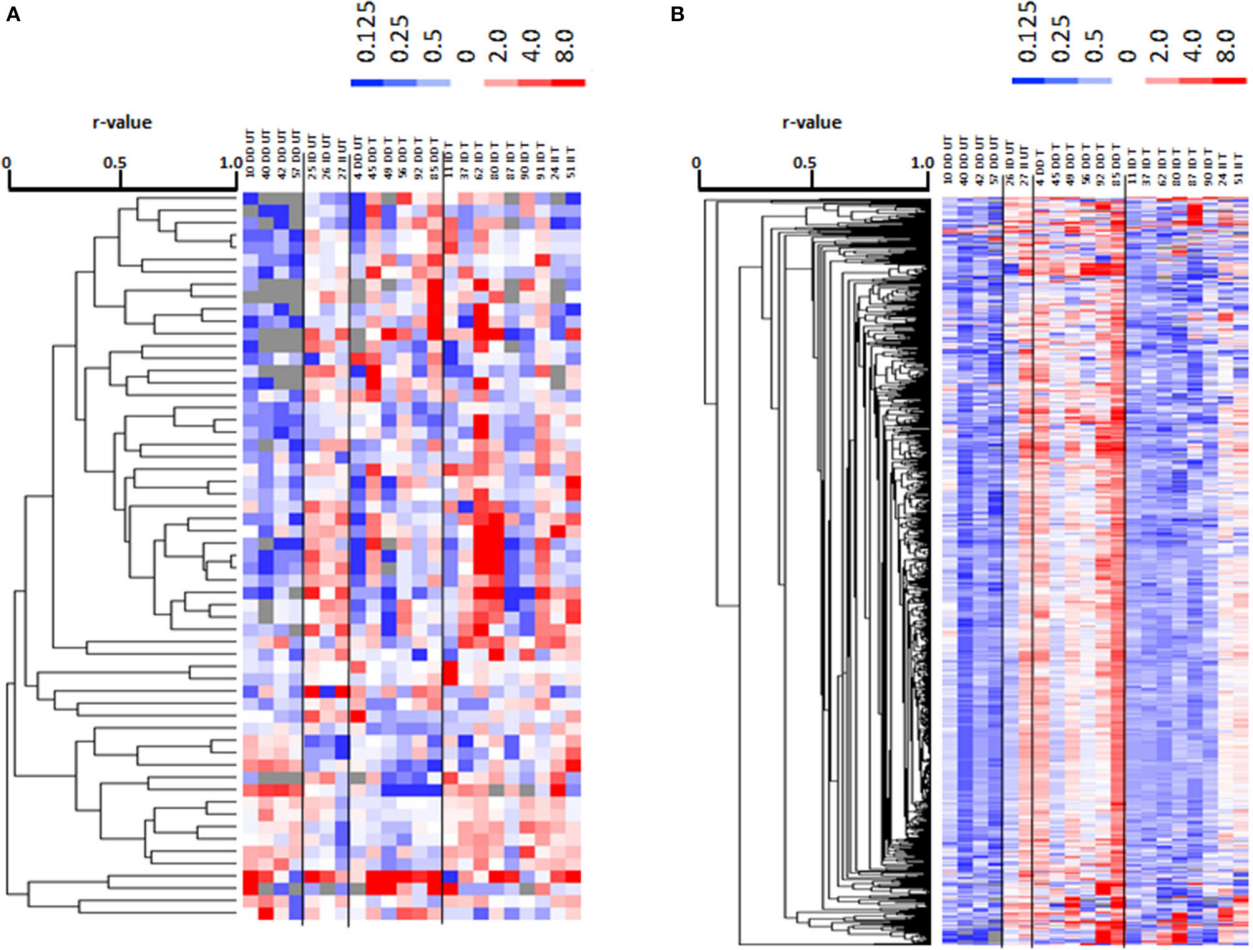
Profiling for ACE-I/D × training dependent metabolites post exercise. Cluster analysis of linear relationships between 59 altered metabolite compounds **(A)** and 780 altered non-polar (lipid) species **(B)** in a subset of subjects (7 untrained and 15 endurance-trained) 30-min post- vs. pre-exercise. Alterations in levels are given in color code (up: red, down: blue) with the scale being indicated to top of each panel. On top the data are labeled by genotype (II, ID; DD) and training status (T, trained; UT, untrained) and subject number. To the right, the compound IDs are indicated according to the human metabolome data base (HMDB). Dendrograms to the left indicates the correlations between compounds. Lines separate profiles according to the I-allele. Unique and identified compound were subsequently identified. Note: A clearly distinguishable pattern of alterations in lipid levels is evident for the trained vs. untrained subjects with the ACE-DD genotype.

Metabolomic profiling identified 924 polar metabolites and 2467 non-polar metabolites (i.e., lipids) in *vastus lateralis* muscle. At rest, the levels of 455 metabolite species differed between trained and untrained subjects, 122 of which were identified. 121 annotated metabolites, comprising prominent metabolites such as acetyl-CoA, carbonic acid, dGTP, D-Mannose, glycerol 3-phosphate, L-aspartic acid, L-glutamic acid, and glutathione, were increasingly abundant in trained subjects (Table S3). One metabolite, 2-methyl-3-pentenoic acid (HMDB31562), was less abundant in *m. vastus lateralis* of the trained subjects. 879 lipidic compounds were less abundant in the *m. vasti* of the trained compared to the untrained subjects, 29 of which were annotated (Table S3). 13 metabolites and 13 non-polar lipids demonstrated an exercise response (Tables S4, S5). 59 metabolites, including 24 uniquely annotated species, and 780 non-polar lipids, including 13 annotated species, demonstrated an exercise response, which varied between ACE I-allele carriers in the trained subjects, and/or untrained subjects (Figure 7, Table 3). Thereby the I-allele related response of lipid compounds post exercise in untrained subjects was inversed in the trained subjects.

**Table 3:** Compounds, which exercise responses demonstrated an effect of ACE-I/D × training state.

**Metabolite compound**	**compound ID**	**class**	**formula**	**name**	**post vs. pre untrained**		**trained**	
					**DD**	**ID/II**	**DD**	**ID/II**
10.76_120.0159 m/z	HMDB03966	alpha amino acids	C5H11NO2Se	Selenomethionine	0.3	0.9	0.6	0.9
7.10_390.1360n	HMDB31422	stilbene glycosides	C20H22O8	cis-Piceid	20.6	32.1	40.7	6.1
9.16_632.1774 m/z	HMDB30585	cyclohexylphenol	C25H24O9	Silymonin	0.1	0.7	1.7	0.7
11.33_101.0596 m/z	HMDB32743	phenolic glycoside	C21H30O13	Phloroacetophenone 6′-[xylosyl-(1->6)-glucoside]	0.0	0.5	1.6	1.0
10.78_120.0153 m/z	HMDB34589	isoflavone	C19H14O10	Shoyuflavone B	0.0	2.0	0.7	1.1
11.17_108.9950 m/z	HMDB15108	lactam	C24H21F2NO3	Ezetimibe	0.1	0.6	1.5	1.2
10.73_250.9593 m/z	HMDB01202	pyrimidine nucleotide	C9H14N3O7P	dCMP	0.3	0.7	0.6	2.0
10.77_94.9788 m/z	HMDB01248	flavin nucleotide	C27H33N9O15P2	FAD	0.1	1.6	0.6	1.7
11.00_94.9787 m/z	HMDB05774	oligopeptide	C32H37N5O5	Endomorphin-2	0.1	1.1	25.7	2.4
6.33_164.0723 m/z	HMDB06344	n-acyl-alpha amino acid	C13H16N2O4	Alpha-N-Phenylacetyl-L-glutamine	0.7	1.5	1.7	2.2
11.70_116.9274 m/z	HMDB28723	peptide	C11H22N6O4	Arginyl-Gamma-glutamate	0.0	1.6	122.6	1.2
9.89_194.9369 m/z	HMDB41757	phenylpropanoid	C22H20O13	Isorhamnetin 4'-O-glucuronide	0.5	0.6	2.3	0.7
9.55_804.1369n	HMDB38493	peptide	C11H19NO7	N-(1-Deoxy-1-fructosyl)proline	0.1	1.3	1.7	3.2
10.43_173.0825 m/z	HMDB34930	sesquiterpenoid	C17H24O4	(3beta, 6beta)-Furanoeremophil-ane-3,6-diol 6-acetate	1.4	1.0	1.0	2.5
10.74_192.0229 m/z	HMDB14823	antibiotic	C20H15F3N4O3	Trovafloxacin	0.3	0.8	0.8	2.6
7.15_338.0347 m/z	HMDB39737	fatty acyl	C17H22O2	(all-E)-1,8,10-Heptadecatriene- 4,6-diyne-3,12-diol	0.2	1.5	1.1	1.3
10.78_126.9047 m/z	HMDB00169	carbohydrate	C6H12O6	D-Mannose	0.8	0.9	0.5	1.1
4.04_316.2614n	HMDB11532	glycerolipid	C18H36O4	MG(0:0/15:0/0:0)	0.8	1.1	1.3	1.9
7.66_130.0632n	HMDB31216	beta-keto acids	C6H10O3	Ethyl acetoacetate	1.6	1.3	0.8	1.4
7.56_159.0170n	HMDB41083	thiolactam	C6H9NS2	(E)-Raphanusanin	0.4	2.1	1.1	20.8
9.11_444.1519n	HMDB14897	antibiotics	C22H24N2O8	Tetracycline	0.3	2.5	0.9	6.8
4.04_302.2457n	HMDB11530	glycerolipid	C17H34O4	MG(0:0/14:0/0:0)	0.4	0.9	2.0	1.4
8.45_342.1188n	HMDB00186	o-glycosyl	C12H22O11	Alpha-Lactose	0.8	2.3	0.6	1.7
8.30_215.0543n	HMDB00114	phosphoramides	C5H14NO6P	Glycerylphosphorylethanolamine	0.7	11.2	1.8	0.8
**Lipid compound**	**compound ID**	**class**	**formula**	**name**	**post vs. pre untrained**		**trained**	
					**DD**	**ID/II**	**DD**	**ID/II**
10.89_358.3090n	4266224	glycerolipid	C21H42O4	MG(18:0/0:0/0:0) [rac]	0.29	1.02	2.66	0.48
11.18_390.2776n	7851039	sterol	C24H38O4	3beta-Hydroxy-7-oxo- 5alpha-cholan-24-oic Acid	0.31	0.87	6.65	1.03
11.61_294.2565n	123060124	fatty acyl	C19H34O2	18:2(5Z,9Z) (16Me)	0.35	1.27	1.07	100.17
11.87_733.5652n	85292011	glycerophospholipid	C40H80NO8P	PE(18:0(10(R)Me)/16:0)	0.61	1.31	0.84	2.53
12.78_426.3868n	123068965	sterol	C30H50O	Xestosterol	0.47	1.29	1.12	0.47
13.26_418.3445n	85300589	sterol	C27H46O3	24R-Cholest-5-en-3beta, 7-alpha,24-triol	0.44	1.19	1.38	0.40
13.37_492.4918n	123060451	fatty acyl	C33H64O2	Heptadecyl palmitoleate	0.43	1.01	5.08	0.59
13.47_532.5234n	135636492	fatty acyl	C36H68O2	Oleyl oleate	0.42	1.03	3.31	0.65
13.50_520.5232n	123060453	fatty acyl	C35H68O2	Nonadecyl palmitoleate	0.44	0.86	3.26	0.67
6.65_302.2208n	74382395	prenol lipid	C20H30O2	Neoabietic acid	0.37	1.03	3.43	0.77
6.79_156.1152n	85291278	fatty acyl	C9H16O2	2,6-Dimethyl-5-heptenoic acid	0.58	1.08	1.45	0.64
7.58_182.1309n	7982525	fatty acyl	C11H18O2	4-undecynoic acid	0.70	1.03	1.55	0.67
8.69_212.1779n	135637980	fatty acyl	C13H24O2	5-Ethylundecane-2,4-dione	0.58	3.73	1.91	0.75

*List of 24 annotated unique metabolites, and 13 lipids, which demonstrated an exercise response, which varied between I-allele carriers in the trained subjects, and/or untrained subjects. Compound ID reflect entries from the human metabolome database (HMDB code; http://www.hmdb.ca/textquery) or substance identifier database (SID code; https://pubchem.ncbi.nlm.nih.gov/substance/)*.

### Interrelationships

A number of relationships were identified between characteristics of muscle composition and performance during one- and two-legged exercise (Figure S1). Peak power output (PPO) during two- and one-legged exercise correlated to CSA of *m. vastus lateralis* (*r* = 0.51, *r* = 0.44) and quadriceps CSA (*r* = 0.64, *r* = 0.59), MCSA of type I (*r* = 0.65, *r* = 0.54) and MCSA of type II muscle fibers (*r* = 0.51, *r* = 0.46), and capillary-to-fiber-ratio (*r* = 0.43, *r* = 0.46). Serum angiotensin 2 concentrations correlated with ACE transcript levels, expression levels of the angiogenic proteins VEGFA and tenascin-C, glycogen concentration and capillary density (Table S6). ACE transcript expression in *m. vastus lateralis* was negatively correlated to angiotensin 2 levels (*r* = −0.92) and positively correlated to mean CSA of type I (*r* = 0.55) and type II muscle fibers (*r* = 0.69).

## Discussion

The renin-angiotensin-aldosterone-system exerts an important role in systemic regulation of blood circulation and blood pressure. Despite paucity for a role in modifying metabolic function and performance of muscle during exercise (Dietze and Henriksen, 2008; Vaughan et al., 2016), this system has not been well characterized at the tissue level during exercise. Substrate delivery and angiogenic factor regulation in exercising muscle are strong candidates for the processes driving local effects of angiotensin with exercise, because the capillary tree becomes increasingly perfused with the onset of contraction (Clifford and Hellsten, 2004; Korthuis, 2011), which may trigger the release of vasoactive and pro-angiogenic factors (such as VEGFA) from the mechanically sheared endothelial wall of perfused blood vessels (Hoier et al., 2013). However, the former processes may be importantly affected by the training status of subjects, because repeated exercise improves the capacity for muscle perfusion through an enhanced capacity for exercise-induced vasodilatation and an increase of the capillary bed (Flück and Hoppeler, 2003; Clifford and Hellsten, 2004; Mortensen et al., 2014). Our previous studies associated the ACE-I allele through a reduction in angiotensin production, with increased capillarisation and improved aerobic substrate pathways with endurance training (Vaughan et al., 2013, 2016). Here we assessed the influence of the prominent insertion/deletion polymorphism in the ACE gene, which entails the production of angiotensin 2 peptide, on muscle composition, capillarisation, metabolites and performance with specific regard on the influence of the training state. Because training typically elevates the capacity for perfusion through an increase in capillarisation and vasodilatation (Flück and Hoppeler, 2003; Clifford and Hellsten, 2004) we hypothesized that training modifies the reported influence of the insertion allele on exercise-induced reactions of metabolites, which are consumed during contraction, muscle capillarisation and expression of pro-angiogenic factors (Flueck et al., 2010; Vaughan et al., 2013, 2016) which explain differences in peak aerobic performance at baseline and with training. Our genetical approach exposes distinct interaction effects of the ACE-I/D genotype and training status regarding exercise-induced alterations of metabolites, the expression of the pro-angiogenic factors VEGFA and tenascin-C, ACE transcript levels, and (fiber) CSAs in *m. vastus lateralis*. By contrast muscle capillarisation in the same muscle and systolic blood pressure, and aerobic power of untrained subjects, were elevated in ACE I-allele carriers, which outweighed the influence of the training state. The observed dependencies, in a muscle being frequently recruited during endurance exercise, highlight a role for genetically modified angiotensin production in metabolic regulation of contracting muscle and the subsequently regulation of “metabolic” plasticity of muscle during recovery from repeated bouts of exercise.

### Systemic relationships

Because the ACE genotype affects systemic aspects of exercise (Hernández et al., 2003), which may be differently influenced by the ACE-I/D genotype, we selected a one-legged exercise stimulus to avoid central limitations in substrate supply to working muscle when exercising at high intensity. Notably, the validity of this assumption has been put into evidence post-hoc put based on the effect of the ACE-I/D genotype on systolic blood pressure (Table S1). The selected one-legged exercise intervention also allowed maximizing the metabolic stimulus for recruited muscle groups. Because perfusion-related events show a rapid onset (Clifford and Hellsten, 2004) we assessed level alterations in VEGFA and tenascin-C protein and muscle metabolites 30 min after exhaustive endurance exercise. Interestingly we identify that non-carriers of the ACE I-allele produced peak power during the one-legged exercise at an elevated fraction of peak aerobic power output as determined during two-legged exercise (i.e., 0.62 vs. 0.58 with a *p*-value of 0.03; see FPPO in Table S1). This difference possibly reflects that the deployed one-leg intervention weighs muscle aspects more than central aspects for power production during cycling type leg endurance exercise. This view is supported by the positive influence of the ACE I-allele and training on the MCSA of type I and II fibers, and capillary-to-fiber ratio in the studied knee extensor muscle. The higher FPPO in ACE-DD genotypes suggest that this genotype demonstrated a certain degree of central “handicap” to allocate resources for muscle-based production of power output under aerobic conditions as previously discussed (Hernández et al., 2003).

The untrained rather than the trained ACE-I-allele carriers, i.e., *p* = 0.005 vs. *p* = 0.744, demonstrated a difference in FPPO between I-allele carriers during two-legged exercise and a trend for an interaction effect between training state and I-allele was seen for FPPO (*p* = 0.089; Table S1). This indicates that local mechanisms were more taxed in the studied non-carriers of the ACE I-allele during extensive exercise and that this influence depended on the training state. Several local muscle mechanisms (including the higher MCSA and capillarisation of muscle fibers (Table S2, Figure 2) may contribute to this elevated aerobic performance. For instance ACE-I-allele dependent differences in the adjustments of the volume density of mitochondria and intramyocellular lipid stores in muscle fibers with endurance training (Vaughan et al., 2013), may contribute to the observed interaction between training state and I-allele for FPPO. Our findings now show that muscle parameters which influence endurance performance with training are already favorably affected by the ACE-I-allele. For instance, glycogen concentration was to a larger degree reduced post exercise in D-allele carriers irrespective to training state whereas muscle capillarisation was higher in ACE I-allele carriers (Table S2, Figures 3A,B, 6). Consistently with the former, we identify that trained ACE-DD genotypes demonstrated a 55 μg mg-1 lower glycogen concentration in *m. vastus lateralis* than the trained ACE-II genotypes, probable reflecting the higher reliance on glycogen during endurance exercise and handicap in the replenishment of these intramyofibrillar carbohydrate stores in ACE-DD genotypes (Vaughan et al., 2016).

A suspicious finding was that rather a small number of metabolite compounds were affected 30-min post exercise when genotype and training status were not considered (Tables S4, S5). Amongst the affected compounds were few that deserve a discussion. For instance, 2-keto glutaramic acid, which is a (neuro)toxic metabolite of transamination reactions involving L-glutamine (Cooper and Kuhara, 2014). It constitutes a critical component of nitrogen metabolism and is detoxified in a reaction with H_2_O to yield oxoglutaric acid (or alpha-keto glutarate), the latter of which is involved in the regulation of protein synthesis (Sahai et al., 1991; Cai et al., 2016). Other exercise-regulated compounds were part of foods, alcohols or medication (Tables S4, S5), including some which reflected antibiotic or anticancer treatment, raising the question whether certain subjects did violate the inclusion criteria.

Overall an important interaction effect between the ACE-I/D genotype and the training state was identified for RERpeak and the concentration of muscle metabolites and lipids, and glycogen 30-min after one-legged exercise. Twenty-four metabolites were identified, which alterations post exercise varied between the trained and untrained subjects in dependence of the ACE I-allele (Figure 7A, Table 3). This included dCMP and FAD, that exert a critical function in cell metabolism and the monoacylglycerides MG(0:0/14:0/0:0) and MG(0:0/15:0/0:0). In this respect, we measured a sizeable number of lipid compounds (i.e., 780), which demonstrated an exercise response that varied between I-allele carriers and training states. Figure 6B illustrates that the regulated compounds strongly differentiated between untrained and trained subjects not carrying the ACE I-allele. Notably there was an important inversion of compound regulation in ACE-DD genotypes. Thirteen of these non-polar compounds were identified as prominent lipid species comprising 7 fatty acyls, two sterols, a glycerolipid and a glycerophospholipid (Table 3). The latter observations relate to identified ACE-I/D dependent expressional regulation of lipid metabolizing enzymes post exercise (i.e., LPL transcript) and exaggerated IMCL accumulation in I-allele carriers after endurance training (Vaughan et al., 2013). Interestingly, the higher post vs. pre-ratios for lipid compounds (Figure 7B) in trained than untrained ACE-DD carriers mirror the changes in RERpeak with training of this genotype (Table S1), which is indicative of a reduced glucose utilization in the trained subjects. While the extent to which the metabolic alterations represents aerobic metabolism of lipids awaits identification through the identification of affected non-polar species, our results emphasize that the ACE I-allele confers a metabolic advantage (i.e., substrate utilization) for endurance performance by affecting muscle metabolism.

We observed a training-state-related inversion of the ACE-I/D-dependent effects of exercise on muscle lipids and metabolites (Table S2). These training-related metabolic differences between genotypes compare to larger differences in capillary-to-fiber ratio between ACE I-allele carriers and non-carriers in trained compared to untrained subjects (Figures 3A,B). The findings indicate that the observed genotype effect on muscle metabolites reflects an altered capacity for capillary perfusion during exercise.

The notion of the influence of the ACE-I/D on the muscle vasculature is supported by I-allele modulated level alterations of the pro-angiogenic protein, VEGFA, 30-min post exercise, irrespective of the training status (Figures 4C,D). VEGFA expression is understood to be secreted from muscle fibers into the interstitium with exercise and to bind and modify blood vessels thereby enhancing their perfusion and proliferation (Mac Gabhann et al., 2006; Hoier et al., 2013).

Respective to angiogenesis, we find that the exercise response of tenascin-C protein levels demonstrates an interaction effect between ACE-I/D genotype and training status (Figures 4E,F). Similar to VEGFA, tenascin-C protein expression in human skeletal muscle is associated mostly with capillary structures and blood vessels in the interstitium, especially endothelial and smooth muscle cells (reviewed by Valdivieso et al., 2017) and is associated with exercise-induced angiogenesis through effects on morphogenesis of blood vessel-associated cells (Valdivieso et al., 2017). Both, VEGFA and tenascin-C expression and/or secretion are affected by angiotensin 2 in different cell types (Hahn et al., 1993; Gruden et al., 1999; Pupilli et al., 1999; Alagappan et al., 2007; Ptasinska-Wnuk et al., 2007) and mediate angiotensin 2-induced vascular reactions (Mackie et al., 1992; Pupilli et al., 1999; Amaral et al., 2001; Ballard et al., 2006). We identified negative correlations between serum angiotensin 2 concentrations and levels of VEGFA and tenascin-C proteins pre- and post-exercise (Table S6). Possibly the observed interaction between ACE-I/D genotype and training status for the exercise response of tenascin-C, especially the 23% reduced levels in untrained ACE-DD genotypes, reflects an angiotensin related vaso-reactive response. In this regard, the interaction effect of the ACE-I/D genotype and training status on capillary-to-fiber ratio in *m. vastus lateralis*, and the correlations between capillary-to-fiber ratio and serum angiotensin 2 concentrations are of interest (Table S6). Collectively, these observations are in line with the suggested role of angiotensin modulated tenascin-C expression, and VEGFA expression, in exercise-induced structural remodeling of capillaries (Mathes et al., 2015; Vaughan et al., 2016). Tenascin-C and VEGFA are to a different extent implied in the regulation of morphogenesis and proliferation which control capillary growth by splitting and sprouting angiogenesis, both of which are induced by exercise (Williams et al., 2006; Valdivieso et al., 2017). The separable effects of the ACE-I/D genotype and training status on post exercise alterations of tenascin-C and VEGFA in muscle therefore suggest that the ACE-I/D genotype and training status may differently modify the splitting and sprouting angiogenesis in exercised muscle.

A role of the local ACE system for capillary reactions to the studied one-legged stimulus is supported by the correlation between capillary density and serum angiotensin 2 concentration post exercise (*r* = 0.91). This emphasizes that the rise in serum angiotensin 2 with exercise (Staessen et al., 1987) is related to the surface area of capillary lumen in muscle where ACE situates and is acutely activated by shear stress (Barauna et al., 2011). In turn, this may lead to release of angiogenic proteins from endothelial cells that lie down-stream, and/or vasoconstriction of downstream vessels through the produced angiotensin 2. The latter contention for a negative feedback mechanism of ACE/angiotensin 2 regulation (Barauna et al., 2011) is supported by the negative correlation between ACE transcript expression and angiotensin 2 levels (*r* = −0.92) before exercise (Table S6); possibly reflecting a negative relationship between the local and systemic regulation of angiotensin 2 production at rest. Intriguingly, ACE activity at rest was not dependent on ACE-I/D genotype (*p* = 0.569) or training (*p* = 0.286), possibly suggesting subtle effects and interferences (Kosunen et al., 1976) which were not controlled in our investigation.

The suggestion of an exercise modulated local ACE system is supported by the interaction effect of ACE-I/D genotype and training status on ACE transcript levels in the studied knee extensor muscle (Table S2, Figure 5). Thereby ACE transcript levels were 28% lower in the untrained ACE I-allele carriers than non-carriers whereas they were 237% higher in the trained ACE I-allele carriers than non-carriers. The observed ACE I-allele dependent up-regulation of the ACE transcripts in trained subjects post exercise (Figure 5, Table S2) suggests that the genetic mechanism, which silences ACE transcript expression, is in part overridden by repeated exercise.

## Limitation

This investigation was performed under several constraints. First, this is a cross-sectional study. Thus, except for the period between the recruitment and the tests, and measures of aerobic capacity we did not control for the lifestyle and training. As well, we identified post-hoc that age differed in association with the ACE-I/D genotype (Table S1). This effect was explained by an average of 8.2 and 8.7 years, respectively, higher age of trained ACE-ID genotypes than ACE-DD (*p* = 0.001) and ACE-II genotypes (*p* = 0.003). Despite the fact, that we did not identify an association between age and training status, performance measure or muscle parameter (Table S7), we cannot exclude the possible role of age as a confounder in our investigation. In line with previous observations, however, we identified effects of the ACE-I/D genotype on muscle composition and found sizable differences in the metabolic and angiogenic response of muscle to the stimulus of exhaustive one-legged exercise. Due to the invasive nature of the biopsy collection, we were ethically bound to use as few subjects as possible. Thus, despite the characterization of the metabolic response in a number of subjects with our invasive approach, we found that the number of homozygous ACE I-allele carriers remained small when considering interactions of the ACE-I/D genotype with training. We therefore primarily addressed the influence of the ACE I-allele on metabolic variables as we previously identified that this allele did produce a dominant effect on muscle parameters (Vaughan et al., 2013, 2016). Regarding the overall concern of the assessed number of subjects we identify (based on effect sizes, data not shown) that observed differences for muscle parameters were considerably larger than what is commonly observed for whole body parameters, thus allowing to expose gene × phenotypic associations with a rather high resolution. Functional relevance of ACE-I allele related differences in muscle structure is pointed out by the observation that peak aerobic power output during one-legged and two-legged exercise was 19% higher in ACE-II than ACE-DD genotypes and peak power output during two-legged exercise remained 14% higher in trained ACE-II than ACE-DD genotypes. Interestingly, interactions between endurance training status and ACE-I/D genotype were identified for muscle-based parameters of muscle performance, such as CSA of *m. vastus lateralis*, MCSA and percentage of the embedded type I muscle fibers, glycogen concentration, and exercise-induced alterations in lipid species and peak respiration exchange ratio (Figure 1). Muscle CSA, muscle fiber MCSA, glycogen content and aerobic combustion of lipids in muscle fibers set the capacity for continued force production (Jones et al., 2004). Our present novel observations therefore indicate that local metabolic effects of regular intense physical activity may override in part the influence of the ACE-I/D genotype on aerobic muscle performance. In this respect the lack of pre- and post- exercise measures on blood flow is a considerable limitation regarding mechanistic conclusions on the contribution of ACE-I/D genotype and training modulated vasodilatation in exercised muscle and warrants further exploration.

## Conclusion

Our investigation documents a higher aerobic performance of untrained ACE I-allele carriers of white British descent, which is related to muscle and muscle fiber CSA, and capillarisation. The observations further emphasize that repeated, intense exercise (training) in part overrides ACE-I/D polymorphism mediated genetic influences at the local muscle level on ACE expression and the downstream angiogenic and metabolic response to endurance exercise; and this is related to the increased muscle capillarisation. The findings motivate further investigations to address the mechanistic contribution of training-induced modifications in angiotensin-controlled capillary perfusion and angiogenic protein secretion in working muscle.

## Author contributions

PV: Performed experiments, prepared figures, edited and revised the manuscript. EL: Performed experiments, analyzed data, edited and revised the manuscript. MB: Performed experiments, analyzed data, prepared figures. SW: Performed experiments, conception and design of research. JR: Performed experiments, edited and revised the manuscript. DV: Performed experiments, conception and design of research, analyzed data. MF: Conception and design of research, performed experiments, analyzed data, interpreted results of experiments, funding, prepared figures, drafted the manuscript, edited and revised the manuscript.

### Conflict of interest statement

The authors declare that the research was conducted in the absence of any commercial or financial relationships that could be construed as a potential conflict of interest.

## Supplementary material

Figure S1 Correlation matrix of for muscle composition and endurance performance. **(A)** *P*-value weighed correlations, i.e., only those relationships with a *p* < 0.02 are shown, are shown in color coding. **(B)** Scatter plots of selected linear relationships demonstrating significant correlations. The line of regression and the 95% confidence interval is shown along with the identified values for r2.

Table S1 Interaction effects of training status and ACE-I/D genotype on physiological characteristics. Values represent mean ± SD of baseline (i.e., pre) values in function of training status and ACE-I/D genotype and the corresponding *p*-values of the (interaction) effects over the 28 endurance-trained and 24 untrained subjects (untrained: 11xACE-DD, 10xACE-ID, 3xACE-II; trained: 15xACE-DD, 10xACE-ID, 3xACE-II) as assessed with univariate ANOVAs. BMI, body mass index; BP, heart pressure; FFM, fat-free mass; PPO1, peak power output during one-legged exercise; PPO, peak power output (during two-legged exercise); FPPO, fraction of PPO at which the one-legged exercise was performed; RERdelta1, difference between peak respiration exchange ratio during one-legged exercise and rest; RERpeak1, peak respiration exchange ratio during one-legged exercise; RERpeak, peak respiration exchange ratio during two-legged exercise; RERrest1, respiration exchange ratio before the one-legged exercise; RERpeak, respiration exchange ratio before two-legged exercise; V°O2peak1, peak oxygen uptake during one-legged exercise; V°O2peak, peak oxygen uptake (during two-legged exercise); V°O2peakr1, body mass-related peak oxygen uptake during one-legged exercise; V°O2peakr, body mass-related peak oxygen uptake during two-legged exercise; FV°O2peak, fraction of V°O2peak at which the one-legged exercise was performed.

Table S2 Interaction effects of training status and ACE-I/D genotype on composition and angiogenic factor expression of skeletal muscle. Values represent mean ± SD of baseline (i.e., pre) values in function of training status and ACE-I/D genotype and the corresponding *p*-values of the (interaction) effects over the 28 endurance-trained and 24 untrained subjects (Univariate ANOVA). ^*^*p* < 0.05 vs. ACE-DD of same training status. ^*$*^*p* < 0.05 vs. untrained of same genotype. CD, capillary density; C:F, capillary-to-fiber ratio; PArea TypeI, percentage of the muscle biopsy CSA being composed of being covered by type I muscle fibers; PTypeI, percentage of type I muscle fibers.

Table S3 Metabolites and lipids, which abundance differed between trained and untrained subjects before exercise.

Table S4 Metabolites (HMDB match) which abundance was affected with exercise.

Table S5 Lipids (LipidMaps match) which abundance was affected with exercise.

Table S6 Correlations to elements of the ACE system in skeletal muscle. Pre and post refer to the time point respective to one-legged exercise.

Table S7 Associations between age and parameters of performance. P-values of Chi-2 based tests between age and the indicated parameter.
