# Supplementary figures and images for: The Metabolic Response of Skeletal Muscle to Endurance Exercise Is Modified by the ACE-I/D Gene Polymorphism and Training State
Source: Front Physiol. 2017 Dec 14;8:993. doi: 10.3389/fphys.2017.00993 (PMC5735290; doi:10.3389/fphys.2017.00993)

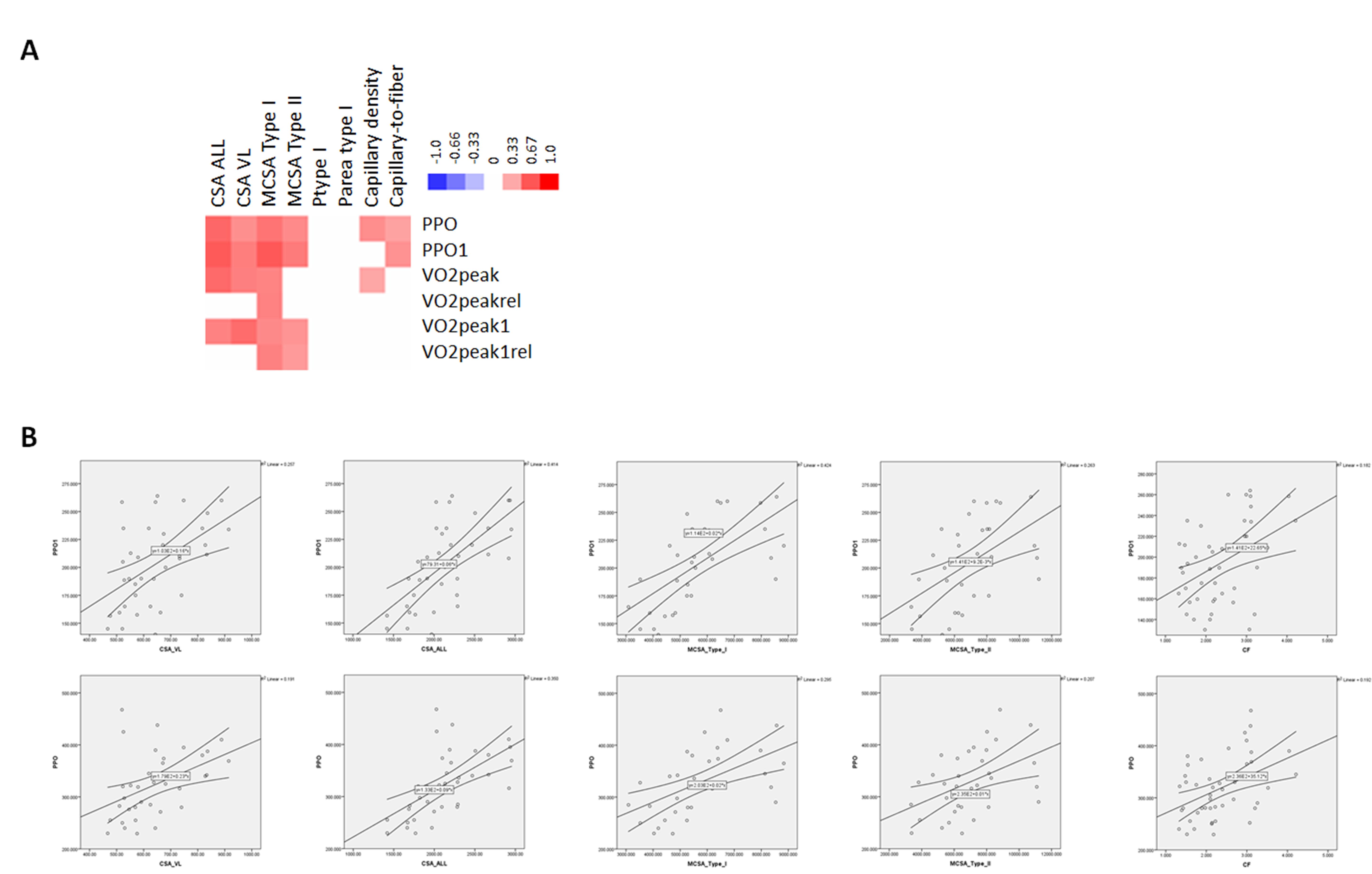

Supplement: Figure S1 — Correlation matrix of for muscle composition and endurance performance. (A) P-value weighed correlations, i.e., only those relationships with a p < 0.02 are shown, are shown in color coding. (B) Scatter plots of selected linear relationships demonstrating significant correlations. The line of regression and the 95% confidence interval is shown along with the identified values for r2. [file Image1.tif]
