# Supplementary material for: The Metabolic Response of Skeletal Muscle to Endurance Exercise Is Modified by the ACE-I/D Gene Polymorphism and Training State
Source: Front Physiol. 2017 Dec 14;8:993. doi: 10.3389/fphys.2017.00993 (PMC5735290; doi:10.3389/fphys.2017.00993)
Supplement: Table S1 — Interaction effects of training status and ACE-I/D genotype on physiological characteristics. Values represent mean ± SD of baseline (i.e., pre) values in function of training status and ACE-I/D genotype and the corresponding p-values of the (interaction) effects over the 28 endurance-trained and 24 untrained subjects (untrained: 11xACE-DD, 10xACE-ID, 3xACE-II; trained: 15xACE-DD, 10xACE-ID, 3xACE-II) as assessed with univariate ANOVAs. BMI, body mass index; BP, heart pressure; FFM, fat-free mass; PPO1, peak power output during one-legged exercise; PPO, peak power output (during two-legged exercise); FPPO, fraction of PPO at which the one-legged exercise was performed; RERdelta1, difference between peak respiration exchange ratio during one-legged exercise and rest; RERpeak1, peak respiration exchange ratio during one-legged exercise; RERpeak, peak respiration exchange ratio during two-legged exercise; RERrest1, respiration exchange ratio before the one-legged exercise; RERpeak, respiration exchange ratio before two-legged exercise; V°O2peak1, peak oxygen uptake during one-legged exercise; V°O2peak, peak oxygen uptake (during two-legged exercise); V°O2peakr1, body mass-related peak oxygen uptake during one-legged exercise; V°O2peakr, body mass-related peak oxygen uptake during two-legged exercise; FV°O2peak, fraction of V°O2peak at which the one-legged exercise was performed. [file Table1.docx]

***Table S1:*** *Interaction effects of training status and ACE-I/D genotype on physiological characteristics.* Values represent mean ± SD of baseline (i.e. pre) values in function of training status and ACE-I/D genotype and the corresponding p-values of the (interaction) effects over the 28 endurance-trained and 24 untrained subjects (untrained: 11xACE-DD, 10xACE-ID, 3xACE-II; trained: 15xACE-DD, 10xACE-ID, 3xACE-II) as assessed with univariate ANOVAs. Abbreviations: BMI, body mass index; BP, heart pressure; FFM, fat-free mass; PPO1, peak power output during one-legged exercise; PPO, peak power output (during two-legged exercise); FPPO, fraction of PPO at which the one-legged exercise was performed; RERdelta1, difference between peak respiration exchange ratio during one-legged exercise and rest; RERpeak1, peak respiration exchange ratio during one-legged exercise; RERpeak, peak respiration exchange ratio during two-legged exercise; RERrest1, respiration exchange ratio before the one-legged exercise; RERpeak, respiration exchange ratio before two-legged exercise; V̇O2peak1, peak oxygen uptake during one-legged exercise; V̇O2peak, peak oxygen uptake (during two-legged exercise); V̇O2peakr1, body mass-related peak oxygen uptake during one-legged exercise; V̇O2peakr, body mass-related peak oxygen uptake during two-legged exercise; FV̇O2peak, fraction of V̇O2peak at which the one-legged exercise was performed.

***untrained or trained (n=52) untrained (n=24) trained (n=28)***

***factor ACE-I/D Mean ± SD Mean ± SD Mean ± SD effect p-value***

***anthropometry***

**Age** DD 25.7 ± 5.3 26.3 ± 5.5 25.2 ± 5.4 training status 0.3968

[years] I-allele 29.9 ± 5.6 25.0 ± 5.0 29.0 ± 5.9 I-allele 0.0085

ID 31.9 ± 4.7 26.8 ± 5.4 33.4 ± 3.5 ACEID 0.0001

II 23.7 ± 2.8  21.5 ± 0.7 24.7 ± 2.9 training status *I-allele 0.1308

training status * ACEID 0.1473

**Weight** DD 74.9 ± 9.3 76.0 ± 9.7 74.0 ± 9.3 training status 0.7145

[kg] I-allele 80.1 ± 10.7 80.5 ± 14.7 80.0 ± 9.6 I-allele 0.0755

ID 79.6 ± 10.9 83.8 ± 17.8 78.6 ± 9.1 ACEID 0.1771

II 81.8 ± 10.4 73.8 ± 0.4 85.8 ± 10.8 training status *I-allele 0.7306

training status * ACEID 0.2199

**Height** DD 178.8 ± 10.3 180.0 ± 10.4 177.5 ± 10.6 training status 0.9104

[cm] I-allele 183.3 ± 11.7 188.3 ± 7.6 181.7 ± 12.5 I-allele 0.1870

ID 183.9 ± 12.0 187.5 ± 9.6 182.9 ± 12.7 ACEID 0.4055

II 181.7 ± 11.7 190.0 ± 1.4 177.5 ± 12.6 training status *I-allele 0.2988

training status * ACEID 0.5392

**BMI** DD 23.5 ± 2.7 23.5 ± 3.3 23.5 ± 2.1 training status 0.4728

[kgm-2] I-allele 24.0 ± 3.5 24.6 ± 4.0 23.3 ± 2.7 I-allele 0.6100

ID 23.7 ± 3.3 25.0 ± 3.9 22.2 ± 1.5 ACEID 0.5825

II 24.9 ± 4.1 23.4 ± 5.1 26.5 ± 3.0 training status *I-allele 0.4913

training status * ACEID 0.0963

**Body fat** DD 17.4 ± 5.9 19.9 ± 6.5 14.4 ± 3.5 training_status 0.0005

[%] I-allele 17.2 ± 8.3 23.8 ± 8.5 15.1 ± 7.3 I-allele 0.3340

ID 14.7 ± 7.8 22.0 ± 14.3 12.8 ± 5.5 ACE_ID 0.3010

II 21.5 ± 7.9 25.6 ± 0.4 19.5 ± 9.3 training_status * Iallele 0.2360

training_status * ACE_ID 0.7010

**Fat-free-mass** DD 63.3 ± 7.5 60.6 ± 6.9 66.6 ± 7.2 training_status 0.0004

[kg] I-allele 66.2 ± 7.3 58.6 ± 5.9 68.8 ± 5.9 I-allele 0.7350

ID 67.5 ± 5.2 62.3 ± 7.0 68.8 ± 4.3 ACE_ID 0.7470

II 64.2 ± 10.1 54.9 ± 0.0 68.8 ± 9.2 training_status * Iallele 0.2030

training_status * ACE_ID 0.1870

**Systolic BP** DD 123.4 ± 5.3 123.7 ± 3.7 123.0 ± 7.3 training status 0.8257

[mmHg] I-allele 124.1 ± 11.5 121.1 ± 13.1 125.9 ± 10.7 I-allele 0.8563

ID 119.3 ± 10.0 114.8 ± 11.4 122.2 ± 8.7 ACEID 0.0128

II 132.2 ± 9.6 133.7 ± 0.9 131.4 ± 12.3 training status *I-allele 0.6807

training status * ACEID 0.3988

**Diastolic BP** DD 73.2 ± 6.5 73.3 ± 4.8 73.0 ± 8.6 training status 0.7864

[mmHg] I-allele 75.2 ± 7.3 72.9 ± 7.6 76.5 ± 7.2 I-allele 0.4950

ID 74.0 ± 6.8 72.6 ± 9.7 74.9 ± 4.8 ACEID 0.5453

II 77.2 ± 8.4 73.5 ± 2.1 79.0 ± 10.1 training status *I-allele 0.6955

training status * ACEID 0.9041

***performance***

***two-legged***

**PPO** DD 299.5 ± 50.9 267.7 ± 32.8 331.4 ± 46.0 training status 0.0001

[W] I-allele 332.0 ± 61.4 312.7 ± 50.6 338.5 ± 64.5 I-allele 0.0141

ID 324.4 ± 62.4 315.0 ± 61.0 327.1 ± 64.8 ACEID 0.0261

II 355.0 ± 56.7 308.0 ± 39.6 378.5 ± 51.3 training status *I-allele 0.9381

training status * ACEID 0.6972

**V̇O2peak** DD 4011.4 ± 668.2 3631.3 ± 368.6 4391.4 ± 694.7 training status 0.0002

[mLO2min-1] I-allele 4203.6 ± 748.3 4034.3 ± 773.6 4260.0 ± 753.7 I-allele 0.2190

ID 4126.3 ± 784.3 4070.9 ± 951.6 4142.2 ± 770.9 ACEID 0.3179

II 4435.4 ± 630.9 3961.1 ± 509.3 4672.6 ± 593.2 training status *I-allele 0.8923

training status * ACEID 0.8474

**V̇O2peakr**  DD 54.3 ± 7.8 50.6 ± 7.4 58.1 ± 6.5 training status 0.0001

[mLO2min-1 I-allele 53.2 ± 9.4 51.2 ± 4.3 53.9 ± 10.6 I-allele 0.7747

kg-1] ID 52.8 ± 9.3 50.0 ± 3.0 53.6 ± 10.4 ACEID 0.9197

II 54.6 ± 10.7 53.6 ± 6.9 55.1 ± 13.2 training status *I-allele 0.2022

training status * ACEID 0.4350

**RERrest** DD 0.78 ± 0.09 0.80 ± 0.09 0.74 ± 0.05 training status 0.1782

[V̇CO2/V̇O2] I-allele 0.75 ± 0.10 0.80 ± 0.06 0.71 ± 0.11 I-allele 0.5156

ID 0.74 ± 0.10 0.80 ± 0.08 0.64 ± 0.06 ACEID 0.5223

II 0.77 ± 0.10 0.82 ± 0.02 0.74 ± 0.12 training status *I-allele 0.5068

training status * ACEID 0.1973

**RERpeak** DD 1.13 ± 0.10 1.16 ± 0.10 1.04 ± 0.05 training status 0.2750

[V̇CO2/V̇O2] I-allele 1.08 ± 0.14 1.11 ± 0.12 1.06 ± 0.16 I-allele 0.8606

ID 1.12 ± 0.11 1.15 ± 0.12 1.06 ± 0.08 ACEID 0.7430

II 1.05 ± 0.16 1.03 ± 0.04 1.06 ± 0.20 training status *I-allele 0.0739

training status * ACEID 0.0302

***one-legged***

**PPO1** DD 187.8 ± 32.6 170.1 ± 21.1 209.1 ± 31.9 training status 0.0004

[W] I-allele 193.5 ± 49.0 193.0 ± 39.6 193.6 ± 52.8 I-allele 0.6014

ID 183.7 ± 50.1 193.3 ± 49.1 180.9 ± 51.8 ACEID 0.0925

II 222.8 ± 33.9 192.5 ± 24.7 238.0 ± 28.2 training status *I-allele 0.7916

training status * ACEID 0.9422

**V̇O2peak1** DD 3350.1 ± 584.4 3155.4 ± 507.6 3583.6 ± 608.9 training status 0.0723

[mLO2min-1] I-allele 3495.0 ± 675.4 3585.4 ± 690.2 3464.9 ± 687.9 I-allele 0.4651

ID 3407.1 ± 732.0 3523.6 ± 838.4 3373.9 ± 730.2 ACEID 0.4059

II 3758.7 ± 406.7 3709.2 ± 476.9 3783.5 ± 444.3 training status *I-allele 0.6357

training status * ACEID 0.8511

**V̇O2peakr1** DD 44.0 ± 5.3 41.7 ± 5.7 46.7 ± 3.3 training status 0.0090

[mLO2min-1 I-allele 43.6 ± 5.7 42.0 ± 5.3 45.6 ± 5.9 I-allele 0.7830

kg-1] ID 42.8 ± 5.9 40.3 ± 3.7 45.8 ± 6.9 ACEID 0.4140

II 46.2 ± 4.7 47.6 ± 6.7 44.9 ± 2.4 training status *I-allele 0.6530

training status * ACEID 0.2130

**FPPO** DD 0.62 ± 0.05 0.64 ± 0.05 0.61 ± 0.04 training status 0.9634

[fraction] I-allele 0.58 ± 0.08 0.62 ± 0.04 0.57 ± 0.08 I-allele 0.0315

ID 0.56 ± 0.08 0.61 ± 0.04 0.55 ± 0.08 ACEID 0.0087

II 0.63 ± 0.03 0.63 ± 0.00 0.63 ± 0.04 training status *I-allele 0.0893

training status * ACEID 0.1887

**FV̇O2peak** DD 0.84 ± 0.09 0.87 ± 0.10 0.80 ± 0.08 training status 0.0058

[fraction] I-allele 0.83 ± 0.08 0.89 ± 0.04 0.81 ± 0.09 I-allele 0.8618

ID 0.82 ± 0.09 0.87 ± 0.01 0.81 ± 0.10 ACEID 0.2970

II 0.85 ± 0.07 0.94 ± 0.00 0.81 ± 0.04 training status *I-allele 0.9090

training status * ACEID 0.9962

**RERrest1**  DD 0.77 ± 0.09 0.75 ± 0.08 0.81 ± 0.12 training status 0.5155

[V̇CO2/V̇O2] I-allele 0.83 ± 0.13 0.80 ± 0.07 0.85 ± 0.16 I-allele 0.2067

ID 0.85 ± 0.14 0.76 ± 0.05 0.91 ± 0.16 ACEID 0.2772

II 0.79 ± 0.11 0.87 ± 0.04 0.75 ± 0.12 training status *I-allele 0.4838

training status * ACEID 0.8155

**RERpeak1** DD 0.90 ± 0.08 0.90 ± 0.08 0.89 ± 0.08 training status 0.8885

[V̇CO2/V̇O2] I-allele 0.93 ± 0.08 0.97 ± 0.03 0.91 ± 0.09 I-allele 0.2259

ID 0.94 ± 0.06 0.97 ± 0.04 0.93 ± 0.06 ACEID 0.3779

II 0.91 ± 0.11 0.98 ± 0.03 0.87 ± 0.13 training status *I-allele 0.6892

training status * ACEID 0.6903
