# Supplementary material for: The Metabolic Response of Skeletal Muscle to Endurance Exercise Is Modified by the ACE-I/D Gene Polymorphism and Training State
Source: Front Physiol. 2017 Dec 14;8:993. doi: 10.3389/fphys.2017.00993 (PMC5735290; doi:10.3389/fphys.2017.00993)
Supplement: Table S2 — Interaction effects of training status and ACE-I/D genotype on composition and angiogenic factor expression of skeletal muscle. Values represent mean ± SD of baseline (i.e., pre) values in function of training status and ACE-I/D genotype and the corresponding p-values of the (interaction) effects over the 28 endurance-trained and 24 untrained subjects (Univariate ANOVA). *p < 0.05 vs. ACE-DD of same training status. $p < 0.05 vs. untrained of same genotype. CD, capillary density; C:F, capillary-to-fiber ratio; PArea TypeI, percentage of the muscle biopsy CSA being composed of being covered by type I muscle fibers; PTypeI, percentage of type I muscle fibers. [file Table2.docx]

***Table S2:*** *Interaction effects of training status and ACE-I/D genotype on composition and angiogenic factor expression of skeletal muscle.* Values represent mean ± SD of baseline (i.e. pre) values in function of training status and ACE-I/D genotype and the corresponding p-values of the (interaction) effects over the 28 endurance-trained and 24 untrained subjects (Univariate ANOVA). *, p<0.05 vs. ACE-DD of same training status. $, p<0.05 vs. untrained of same genotype. Abbreviations: CD, capillary density; C:F, capillary-to-fiber ratio; PArea TypeI, percentage of the muscle biopsy CSA being composed of being covered by type I muscle fibers; PTypeI, percentage of type I muscle fibers.

***untrained or trained (n=52) untrained (n=18) trained (n=34)***

***factor ACE-I/D Mean ± SD Mean ± SD Mean ± SD effect p-value***

***cross sectional area***

**quadriceps CSA** DD 2077.47 ± 426.44 1867.43 ± 296.66 2329.51 ± 432.85 training status 0.051

I-allele 2292.89 ± 410.77 2264.04 ± 448.18 2311.24 ± 406.66 I-allele 0.143

ID 2200.15 ± 420.47 2085.84 ± 463.66 2257.30 ± 417.71 ACEID 0.121

II 2478.37 ± 350.46 2501.66 ± 363.20 2455.09 ± 416.55 training status *I-allele 0.109

ALL 2174.41 ± 428.08 2013.55 ± 399.21 2319.94 ± 408.76 training status * ACEID 0.309

**vastus lateralis CSA** DD 639.38 ± 126.81 571.14 ± 62.77 721.28 ± 138.00 training status 0.134

I-allele 677.85 ± 124.94 700.21 ± 133.31 663.62 ± 123.69 I-allele 0.342

ID 660.48 ± 149.58 673.97 ± 182.73 653.74 ± 143.86 ACEID 0.475

II 712.59 ± 40.90 735.21 ± 4.15 689.96 ± 51.26 training status *I-allele 0.016

ALL 656.69 ± 125.85 618.69 ± 111.46 691.08 ± 130.73 training status * ACEID 0.075

***Compostion of vastus lateralis***

**capillary density** DD 272.58 ± 32.20 268.57 ± 40.58 276.59 ± 22.05 training status 0.248

I-allele 312.18 ± 38.31 304.16 ± 46.91 320.20 ± 26.99 I-allele 0.001

ID 313.86 ± 43.35 307.59 ± 54.48 320.12 ± 30.61 ACEID 0.002

II 307.15 ± 17.95 293.88 ± 4.43 320.43 ± 16.04 training status *I-allele 0.698

ALL 292.38 ± 40.32 286.37 ± 46.59 298.40 ± 32.82 training status * ACEID 0.855

**capillary-to-fiber** DD 1.99 ± 0.52 1.77 ± 0.41 2.22 ± 0.54 training status 0.002

I-allele 2.67 ± 0.73 2.34 ± 0.61 3.00 ± 0.72 I-allele 0.001

ID 2.73 ± 0.80 2.41 ± 0.68 3.04 ± 0.82 ACEID 0.001

II 2.50 ± 0.48 2.12 ± 0.26 2.88 ± 0.29 training status *I-allele 0.535

ALL 2.33 ± 0.71 2.05 ± 0.58 2.61 ± 0.74 training status * ACEID 0.804

**MCSA Type I** DD 4930.13 ± 991.09 4542.38 ± 885.06 5317.88 ± 977.87 training status 0.001

I-allele 6420.06 ± 1648.13 4913.93 ± 953.19 7378.51 ± 1214.18 I-allele 0.001

ID 6221.65 ± 1742.20 4319.27 ± 773.95 7172.83 ± 1187.83 ACEID 0.001

II 6816.89 ± 1508.16 5706.80 ± 420.76 7926.98 ± 1346.25 training status *I-allele 0.018

ALL 5635.89 ± 1523.43 4695.37 ± 903.81 6397.26 ± 1509.81 training status * ACEID 0.025

**MCSA Type II** DD 5782.62 ± 1453.27 5322.33 ± 1602.95 6242.91 ± 1190.96 training status 0.006

I-allele 8114.10 ± 1784.99 6936.64 ± 977.24 8863.39 ± 1804.28 I-allele 0.001

ID 7812.10 ± 1819.37 6273.76 ± 498.78 8581.28 ± 1751.16 ACEID 0.001

II 8718.09 ± 1701.20 7820.48 ± 664.26 9615.70 ± 2092.08 training status *I-allele 0.308

ALL 6887.00 ± 1985.01 5987.04 ± 1572.93 7615.54 ± 2016.05 training status * ACEID 0.443

**PType I** DD 0.43 ± 0.08 0.42 ± 0.06 0.43 ± 0.10 training status 0.435

I-allele 0.41 ± 0.11 0.39 ± 0.14 0.42 ± 0.08 I-allele 0.219

ID 0.41 ± 0.09 0.47 ± 0.13 0.39 ± 0.05 ACEID 0.592

II 0.40 ± 0.15 0.28 ± 0.08 0.52 ± 0.06 training status *I-allele 0.325

ALL 0.42 ± 0.09 0.41 ± 0.10 0.43 ± 0.09 training status * ACEID 0.024

**PArea Type I** DD 0.39 ± 0.08 0.39 ± 0.08 0.39 ± 0.08 training status 0.525

I-allele 0.36 ± 0.12 0.32 ± 0.15 0.38 ± 0.09 I-allele 0.508

ID 0.36 ± 0.10 0.38 ± 0.15 0.35 ± 0.08 ACEID 0.782

II 0.35 ± 0.15 0.24 ± 0.14 0.47 ± 0.03 training status *I-allele 0.596

ALL 0.37 ± 0.10 0.36 ± 0.11 0.38 ± 0.08 training status * ACEID 0.001

**glycogen** DD 0.09 ± 0.05 0.10 ± 0.06 0.07 ± 0.02 training status 0.802

[mg mg-1] I-allele 0.07 ± 0.05 0.06 ± 0.04 0.09 ± 0.05 I-allele 0.385

ID 0.07 ± 0.05 0.06 ± 0.04 0.08 ± 0.05 ACEID 0.388

II 0.09 ± 0.05 0.05 ± 0.04 0.13 ± 0.02 training status *I-allele 0.027

ALL 0.08 ± 0.05 0.08 ± 0.05 0.08 ± 0.04 training status * ACEID 0.027

***angiogenic factors***

**ACEmRNA** DD 53.08 ± 36.30 79.91 ± 40.57 35.19 ± 18.86 training status 0.597

I-allele 85.02 ± 65.02 57.76 ± 29.39 118.33 ± 81.80 I-allele 0.048

ID 87.89 ± 69.06 59.00 ± 26.23 116.79 ± 87.31 ACEID 0.146

II 73.52 ± 52.08 54.46 ± 43.47 130.69 ± training status *I-allele 0.001

ALL 69.05 ± 54.44 67.08 ± 35.30 70.83 ± 68.19 training status * ACEID 0.006

**ACE_activity** DD 283.86 ± 137.74 318.63 ± 165.90 254.87 ± 117.08 training status 0.286

I-allele 278.27 ± 90.95 300.22 ± 103.97 265.10 ± 85.25 I-allele 0.929

ID 284.10 ± 76.73 334.71 ± 67.75 247.94 ± 63.84 ACEID 0.569

II 283.19 ± 115.68 261.25 ± 122.33 305.13 ± 130.55 training status *I-allele 0.755

ALL 283.82 ± 107.13 311.58 ± 118.64 261.27 ± 94.64 training status * ACEID 0.194

**Tenascin-C** DD 1.44 ± 0.42 1.50 ± 0.29 1.39 ± 0.51 training status 0.243

I-allele 1.74 ± 1.69 2.13 ± 2.19 1.28 ± 0.62 I-allele 0.536

ID 1.76 ± 1.95 2.32 ± 2.48 1.04 ± 0.55 ACEID 0.846

II 1.68 ± 0.37 1.47 ± 0.30 1.89 ± 0.33 training status *I-allele 0.374

ALL 1.61 ± 1.30 1.89 ± 1.73 1.33 ± 0.56 training status * ACEID 0.268

**VEGFA** DD 0.91 ± 0.55 0.96 ± 0.73 0.88 ± 0.38 training status 0.482

I-allele 1.76 ± 0.55 1.84 ± 0.66 1.67 ± 0.41 I-allele 0.001

ID 1.84 ± 0.50 1.94 ± 0.62 1.71 ± 0.30 ACEID 0.001

II 1.53 ± 0.69 1.51 ± 0.81 1.56 ± 0.72 training status *I-allele 0.781

ALL 1.40 ± 0.69 1.50 ± 0.80 1.29 ± 0.56 training status * ACEID 0.852
