# Supplementary material for: The Metabolic Response of Skeletal Muscle to Endurance Exercise Is Modified by the ACE-I/D Gene Polymorphism and Training State
Source: Front Physiol. 2017 Dec 14;8:993. doi: 10.3389/fphys.2017.00993 (PMC5735290; doi:10.3389/fphys.2017.00993)
Supplement: Table S3 — Metabolites and lipids, which abundance differed between trained and untrained subjects before exercise. [file Table3.docx]

***Table S3:*** *Metabolites and lipids, which abundance differed between trained and untrained subjects before exercise*

***Description Compound ID Formula T vs UT q-value(%)***

***metabolites***

2-Methyl-3-pentenoic acid HMDB31562 C6H10O2 0.4 0.0

(-)-Thebaine HMDB29378 C19H21NO3 1.2 0.0

(¬±)-Leptophos HMDB31798 C13H10BrCl2O2PS 1.5 0.0

(3beta,6beta)-Furanoeremophilane-3,6-diol 6-acetate HMDB34930 C17H24O4 1.2 0.0

(4-Hydroxy-3-methoxyphenyl)ethanol HMDB38925 C9H12O3 1.4 0.0

(all-E)-1,8,10-Heptadecatriene-4,6-diyne-3,12-diol HMDB39737 C17H22O2 1.2 0.0

(E)-2-Propenyl [3-(2-propenylthio)-2-propenyl] sulfate HMDB39464 C9H14O4S2 2.2 0.0

(E)-Raphanusanin HMDB41083 C6H9NS2 1.3 0.0

(R)-2-Hydroxyhexadecanoic acid HMDB31057 C16H32O3 1.2 0.0

(S)-3,4-Dihydroxybutyric acid HMDB00337 C4H8O4 1.0 0.0

(S)-Argpyrimidine HMDB37180 C11H18N4O3 1.3 0.0

1,2,10-Trihydroxydihydro-trans-linalyl oxide 7-O-beta- HMDB33237 C16H30O10 2.7 0.0

D-glucopyranoside

1,2,3,4-Tetramethoxy-5-(2-propenyl)benzene HMDB34046 C13H18O4 1.1 0.0

1,6-Dihydroxy-3-methoxy-2-prenylxanthone HMDB34015 C19H18O5 5.9 0.0

1-[(2,5-Dimethylphenyl)azo]-2-naphthalenol HMDB32889 C18H16N2O 2.0 0.0

1-Naphthaleneacetic acid HMDB32708 C12H10O2 1.1 0.0

1-Octene HMDB32449 C8H16 1.2 0.0

2,2-Dihydroperoxypropane HMDB34260 C3H8O4 8.9 0.0

2-Aminomuconic acid HMDB01241 C6H7NO4 2.4 0.0

2-Fucosyllactose HMDB02098 C18H32O15 1.1 0.0

3,7,7,10-Tetramethyl-12-thiabicyclo[9.1.0]dodeca-3,7-diene HMDB36469 C15H24S 1.0 0.0

3-Galactosyllactose HMDB06599 C18H32O16 1.4 0.0

3-Oxoglutaric acid HMDB13701 C5H6O5 1.5 0.0

4-Hydroxy-2-oxoglutaric acid HMDB02070 C5H6O6 1.5 0.0

4-Hydroxybenzyl isothiocyanate HMDB32580 C8H7NOS 1.5 0.0

4-Hydroxyphenylacetonitrile triacetylrhamnoside HMDB32809 C20H23NO8 1.2 0.0

4-O-Methylgallic acid HMDB13198 C8H8O5 1.0 0.0

5-Hydroxy-2-furoic acid HMDB59784 C5H4O4 1.5 0.0

5-O-Galloylhamamelofuranose HMDB39366 C13H16O10 1.2 0.0

5-Phosphoribosylamine HMDB01128 C5H12NO7P 1.3 0.0

5-Sulfosalicylic acid HMDB11725 C7H6O6S 1.1 0.0

Acetyl-CoA HMDB01206 C23H38N7O17P3S 1.5 0.0

Alanyl-Glycine HMDB28687 C5H10N2O3 1.1 0.0

Alanyl-Histidine HMDB28689 C9H14N4O3 1.2 0.0

Alpha-Lactose HMDB00186 C12H22O11 1.7 0.0

Alpha-N-Phenylacetyl-L-glutamine HMDB06344 C13H16N2O4 1.2 0.0

Amritoside HMDB34258 C26H26O18 1.2 0.0

Arginyl-Gamma-glutamate HMDB28723 C11H22N6O4 1.0 0.0

Artemether HMDB15643 C16H26O5 1.0 0.0

Astemizole HMDB14775 C28H31FN4O 1.3 0.0

Avenalumin II HMDB38955 C17H13NO3 1.5 0.0

Beta-Guanidinopropionic acid HMDB13222 C4H9N3O2 1.2 0.0

C.I. Acid Blue 3 HMDB37612 C27H32CaN2O6S2 3.2 0.0

Carbadox HMDB31762 C11H10N4O4 1.1 0.0

Carbonic acid HMDB03538 CH2O3 1.7 0.0

Carboxyifosfamide HMDB60692 C7H15Cl2N2O4P 1.0 0.0

Caryoptosidic acid HMDB34249 C16H24O11 3.9 0.0

CDP-Ethanolamine HMDB01564 C11H20N4O11P2 1.3 0.0

Chrysoeriol 4',7-diglucuronide HMDB39931 C28H28O18 1.5 0.0

Citrusin E HMDB39234 C17H24O9 1.3 0.0

Clofazimine HMDB14983 C27H22Cl2N4 1.4 0.0

CMP-N-trimethyl-2-aminoethylphosphonate HMDB60072 C14H27N4O10P2 1.4 0.0

Cyclodopa glucoside HMDB29833 C15H19NO9 1.5 0.0

Deoxycytidine HMDB00014 C9H13N3O4 1.8 0.0

dGTP HMDB01440 C10H16N5O13P3 1.0 0.0

Diazepam HMDB14967 C16H13ClN2O 1.3 0.0

Diosbulbinoside D HMDB30084 C25H30O11 1.2 0.0

Dithionous acid HMDB59919 H2O4S2 1.0 0.0

D-Mannose HMDB00169 C6H12O6 2.0 0.0

Dulxanthone A HMDB31992 C19H18O6 1.8 0.0

Endomorphin-2 HMDB05774 C32H37N5O5 1.2 0.0

Ethoxyacetic acid HMDB31212 C4H8O3 3.4 0.0

fluorobenzoylpropionic acid HMDB60951 C10H8F2O3 1.4 0.0

Formaldehyde HMDB01426 CH2O 10.8 0.0

Fucose 1-phosphate HMDB01265 C6H13O8P 1.1 0.0

Gestrinone HMDB02720 C21H24O2 1.6 0.0

Glucosamine-1P HMDB01109 C6H14NO8P 1.0 0.0

Glucosylisomaltol HMDB38341 C12H16O8 1.7 0.0

Glutaminyl-Serine HMDB28806 C8H15N3O5 1.7 0.0

Glutaric acid HMDB00661 C5H8O4 1.8 0.0

Glutathione HMDB00125 C10H17N3O6S 1.8 0.0

Glycerol 3-phosphate HMDB00126 C3H9O6P 1.4 0.0

Glyoxylic acid HMDB00119 C2H2O3 1.2 0.0

Hydroxyethyl glycine HMDB61148 C4H9NO3 1.0 0.0

Ibopamine HMDB41906 C17H25NO4 1.4 0.0

Imidazolelactic acid HMDB02320 C6H8N2O3 1.1 0.0

Irinotecan HMDB14900 C33H38N4O6 1.1 0.0

Isoplumbagin HMDB35291 C11H8O3 2.9 0.0

Isopropyl beta-D-glucoside HMDB32705 C9H18O6 1.1 0.0

L-Aspartic acid HMDB00191 C4H7NO4 1.4 0.0

L-Cyclo(alanylglycyl) HMDB31547 C5H8N2O2 1.8 0.0

Lentinic acid HMDB38385 C12H22N2O10S4 1.2 0.0

L-Glutamic acid HMDB00148 C5H9NO4 1.3 0.0

LysoPE(20:0/0:0) HMDB11511 C25H52NO7P 1.1 0.0

Malvidin 3-galactoside HMDB38010 C23H25O12 1.8 0.0

Methyl dodecanoate HMDB31018 C13H26O2 1.1 0.0

Methylhippuric acid HMDB00859 C10H11NO3 1.0 0.0

Methylscopolamine HMDB14605 C18H24BrNO4 1.4 0.0

MG(0:0/15:0/0:0) HMDB11532 C18H36O4 1.3 0.0

N-(2-hydroxymethyl-3-chloro-4-hydroxyphenyl)anthranilic acid HMDB60007 C14H12ClNO4 2.0 0.0

N,N'-dinitrosopiperazine HMDB41941 C4H8N4O2 3.2 0.0

N-[4'-hydroxy-(E)-cinnamoyl]-L-aspartic acid HMDB29234 C13H13NO6 1.7 0.0

N-Acetylaspartylglutamic acid HMDB01067 C11H16N2O8 1.2 0.0

N-Acetylglutamine HMDB06029 C7H12N2O4 1.3 0.0

Neobifurcose HMDB38870 C24H42O21 1.3 0.0

Nicotinamide N-oxide HMDB02730 C6H6N2O2 1.5 0.0

N-Nitroso-pyrrolidine HMDB31642 C4H8N2O 1.2 0.0

Norfuraneol HMDB31859 C5H6O3 22.2 0.0

NPC HMDB60499 C28H30N4O6 1.4 0.0

O-Ureidohomoserine HMDB12271 C5H11N3O4 1.1 0.0

Oxidized glutathione HMDB03337 C20H32N6O12S2 1.4 0.0

Pseudomonine HMDB41438 C16H18N4O4 1.5 0.0

Pyruvaldehyde HMDB01167 C3H4O2 1.0 0.0

Quercetin 7-glucuronide 3-rhamnoside HMDB36264 C27H28O17 1.4 0.0

Quinupristin HMDB15455 C53H67N9O10S 1.0 0.0

Rosoxacin HMDB14955 C17H14N2O3 1.4 0.0

Saccharopine HMDB00279 C11H20N2O6 1.0 0.0

Selenomethionine HMDB03966 C5H11NO2Se 1.8 0.0

Sildenafil HMDB05039 C22H30N6O4S 1.1 0.0

S-Lactoylglutathione HMDB01066 C13H21N3O8S 1.2 0.0

SN38 glucuronide HMDB60511 C28H28N2O11 1.1 0.0

Sulfacytine HMDB15412 C12H14N4O3S 1.5 0.0

Sulfisoxazole HMDB14408 C11H13N3O3S 1.1 0.0

Taurine HMDB00251 C2H7NO3S 1.3 0.0

Tetracycline HMDB14897 C22H24N2O8 1.3 0.0

Thiamine monophosphate HMDB02666 C12H17N4O4PS 1.0 0.0

Thiodiacetic acid HMDB42032 C4H6O4S 1.1 0.0

trans-3,3',4',5,5',7-Hexahydroxyflavanone HMDB30835 C15H12O8 1.4 0.0

UDP-L-iduronate HMDB12304 C15H22N2O18P2 1.2 0.0

UDP-N-acetyl-D-mannosamine HMDB13112 C17H27N3O17P2 1.6 0.0

Wistin HMDB30869 C23H24O10 1.0 0.0

Xanthotoxol glucoside HMDB38626 C17H16O9 1.1 0.0

***Description Compound ID Formula T vs UT q-value(%)***

***lipids***

MG(18:0/0:0/0:0)[rac] 4266224 C21H42O4 0.7 0.0

MG(20:0/0:0/0:0)[rac] 4266228 C23H46O4 0.6 0.0

18:1(7Z)(17Me) 123060132 C19H36O2 0.8 0.0

18:2(5Z,9Z)(16Me) 123060124 C19H34O2 0.5 0.0

PC(14:1(9Z)/20:0) 123060685 C42H82NO8P 0.8 0.0

PC(P-16:0/20:4(5Z,8Z,11Z,14Z)) 7983898 C44H80NO7P 0.8 0.0

PE(18:0(10(R)Me)/16:0) 85292011 C40H80NO8P 0.8 0.0

PE(P-16:0/20:4(5Z,8Z,11Z,14Z)) 123062680 C41H74NO7P 0.8 0.0

PI(18:4(6Z,9Z,12Z,15Z)/20:0) 123065431 C47H83O13P 0.9 0.0

PS(17:2(9Z,12Z)/18:0) 123062953 C41H76NO10P 0.6 0.0

PS(20:1(11Z)/16:0) 123063199 C42H80NO10P 0.5 0.0

(4E,8E,d18:2) sphingosine 74382659 C18H35NO2 0.6 0.0

9E-Hexadecenyl acetate 135636686 C18H34O2 0.6 0.0

3-oxo-eicosanoic acid 7982760 C20H38O3 0.7 0.0

3beta-Hydroxy-7-oxo-5alpha-cholan-24-oic Acid 7851039 C24H38O4 0.7 0.0

Docosanedioic acid 14710836 C22H42O4 0.7 0.0

20S-hydroxycholesterol 49703664 C27H46O2 0.6 0.0

dihydrotachysterol3 / (5E)-(10S)-10,19-dihydrovitamin D3 / 14715218 C27H46O 0.8 0.0

(5E)-(10S)-10,19-dihydrocholecalciferol

pentadecyl hexanoate 135636782 C21H42O2 0.6 0.0

3beta,9alpha-Dihydroxy-11-oxo-5beta-cholan-24-oic Acid 7851178 C24H38O5 0.7 0.0

Dichotellate A 123060263 C26H42O4 1.0 0.0

dihydroxy-fumaric acid 85291357 C4H4O6 1.0 0.0

4'-Methoxychalcone 74380798 C16H14O2 0.8 0.0

Sativic acid 135636132 C18H36O6 0.6 0.0

12-hydroxyjasmonic acid 85291455 C12H18O4 1.0 0.0

2,6-Dimethyl-1,8-octanedioic acid 135636040 C10H18O4 1.0 0.0

Tridecanedioic acid 14710813 C13H24O4 0.9 0.0

beta-santalol 85297270 C15H24O 1.0 0.0

5-Ethylundecane-2,4-dione 135637980 C13H24O2 1.0 0.0
