# Supplementary material for: The Metabolic Response of Skeletal Muscle to Endurance Exercise Is Modified by the ACE-I/D Gene Polymorphism and Training State
Source: Front Physiol. 2017 Dec 14;8:993. doi: 10.3389/fphys.2017.00993 (PMC5735290; doi:10.3389/fphys.2017.00993)
Supplement: Table S4 — Metabolites (HMDB match) which abundance was affected with exercise. [file Table4.docx]

***Table S4:*** *Metabolites (HMDB match) which abundance was affected with exercise*

***Description origin Compound ID Formula Neutral mass (Da) Fold difference q-value(%)***

***[post vs pre]***

Glutathione detoxification system HMDB00125 C10H17N3O6S 307.0848372 3.2 0.0

Saccharopine degradation of lysine HMDB00279 C11H20N2O6 276.1343693 5.9 0.0

2-Keto-glutaramic acid metabolite of glutamine HMDB01552 C5H7NO4 145.0376366 3.3 0.0

Astemizole Antihistamine HMDB14775 C28H31FN4O 458.2458239 0.7 0.0

Sulfacytine bacteriostatic antibiotics HMDB15412 C12H14N4O3S 294.0737604 16.3 0.0

Quinupristin Antibiotics HMDB15455 C53H67N9O10S 1021.474489 2.0 0.0

Silymonin Coffee HMDB30585 C25H24O9 468.1417372 0.8 0.0

Isopropyl beta-D-glucoside herbs and spices HMDB32705 C9H18O6 222.1115493 2.1 0.0

(E)-2-Glucosyl-3,4',5- alcoholic beverages HMDB36294 C20H22O8 390.1360138 21.7 0.0

trihydroxystilbene

Ibopamine drug against heart failure HMDB41906 C17H25NO4 307.1751148 1.9 0.0

Dithionous acid bleaching agent HMDB59919 H2O4S2 129.9374 1.5 0.0

2-Bromoacetaldehyde Organobromides HMDB60344 C2H3BrO 121.9387399 48.2 0.0

SN38 glucuronide cancer drug HMDB60511 C28H28N2O11 568.1650533 1.9 0.0
