# Supplementary material for: The Metabolic Response of Skeletal Muscle to Endurance Exercise Is Modified by the ACE-I/D Gene Polymorphism and Training State
Source: Front Physiol. 2017 Dec 14;8:993. doi: 10.3389/fphys.2017.00993 (PMC5735290; doi:10.3389/fphys.2017.00993)
Supplement: Table S5 — Lipids (LipidMaps match) which abundance was affected with exercise. [file Table5.docx]

***Table S5:*** *Lipids (LipidMaps match) which abundance was affected with exercise*

***Description class Compound ID Formula Neutral mass (Da) Fold difference q-value(%)***

***[post vs pre]***

(4E,8E,d18:2) sphingosine Sphingolipids 74382659 C18H35NO2 297.267 4.3 0.0

2,6-Dimethyl-1,8-octanedioic acid Fatty Acyls 135636040 C10H18O4 202.123 2.3 0.0

Axillarenic acid Fatty Acyls 123060238 C24H46O4 398.340 3.1 0.0

Type IV cyanolipid eicosanoyl ester Fatty Acyls 49703481 C25H45NO2 391.345 139286.4 0.0

Syzygiol Polyketides 74381027 C18H18O5 314.116 2489.9 0.9

Sugeonyl acetate Prenol Lipids 123067406 C17H24O3 276.173 1.2 0.9

1,3Z,6Z,9Z-Heneicosatetraene Fatty Acyls 135637230 C21H36 288.281 0.6 0.0

Dichotellate A Fatty Acyls 123060263 C26H42O4 418.309 0.8 0.0

3,11-dihydroxy myristoic acid Fatty Acyls 7850178 C14H28O4 260.199 0.8 0.0

5E-Dodecenyl acetate Fatty Acyls 135636564 C14H26O2 226.194 0.6 0.0

(4R,6R)-cis-Carveol Prenol Lipids 74382177 C10H16O 152.120 0.7 0.0

3beta-Hydroxy-7-oxo-5alpha-cholan- Sterol Lipids 7851039 C24H38O4 390.278 0.8 0.0

24-oic Acid

Prosopinine Sphingolipids 74382698 C16H33NO3 287.247 0.8 0.0
