# Supplementary material for: The Metabolic Response of Skeletal Muscle to Endurance Exercise Is Modified by the ACE-I/D Gene Polymorphism and Training State
Source: Front Physiol. 2017 Dec 14;8:993. doi: 10.3389/fphys.2017.00993 (PMC5735290; doi:10.3389/fphys.2017.00993)
Supplement: Table S6 — Correlations to elements of the ACE system in skeletal muscle. Pre and post refer to the time point respective to one-legged exercise. [file Table6.DOCX]

***Table S6:*** *Correlations to elements of the ACE system in skeletal muscle.* Pre and post refer to the time point respective to one-legged exercise.

***factors r-value p-value***

angiotensin 2 (pre) : ACE transcript (pre) -0.92 0.001

angiotensin 2 (pre) : tenascin-c (pre) -0.61 0.004

angiotensin 2 (post) : tenascin-c (post) 0.97 0.001

angiotensin 2 (pre) : VEGF (pre) -0.61 0.004

angiotensin 2 (pre) : VEGF (post) -0.81 0.001

angiotensin 2 (pre) : capillary-to-fiber ratio 0.63 0.009

angiotensin 2 (pre) : glycogen (post) 0.84 0.001

angiotensin 2 (post) : glycogen (pre) 0.86 0.001

angiotensin 2 (post) : capillary density 0.95 0.001

ACE transcript (pre) : MCSA type I 0.55 0.002

ACE transcript (pre) : MCSA type II 0.69 0.001

PPO-1 : MCSA type I 0.65 0.001

PPO-1 : MCSA type II 0.51 0.002
