# Supplementary material for: The Metabolic Response of Skeletal Muscle to Endurance Exercise Is Modified by the ACE-I/D Gene Polymorphism and Training State
Source: Front Physiol. 2017 Dec 14;8:993. doi: 10.3389/fphys.2017.00993 (PMC5735290; doi:10.3389/fphys.2017.00993)
Supplement: Table S7 — Associations between age and parameters of performance. P-values of Chi-2 based tests between age and the indicated parameter. [file Table7.DOCX]

***Table S7:*** *Associations between age and parameters of performance.* P-values of Chi-2 based tests between age and the indicated parameter.

***association with age p-value***

training status 0.469

weight 0.170

height 0.032

BMI 0.268

% body fat 0.234

fat-free-mass 0.262

diastolic blood pressure 0.201

systolic blood pressure 0.315

PPO 0.187

VO2peak 0.268

VO2peakr 0.110

RERrest 0.160

RERmax 0.365

PPO1 0.050

VO2peak1 0.267

VO2peak1r 0.130

FPPO 0.644

FVO2 0.248

RERrest1 0.291

RERmax1 0.056

quadriceps CSA 0.261

vastus lateralis CSA 0.261

capillary density 0.150

capillary-to-fiber 0.151

MCSA Type I 0.258

MCSA Type II 0.258

Ptype I 0.258

Parea Type I 0.244

ACE transcript (pre) 0.272

ACE activity (pre) 0.263

glycogen (pre) 0.092

VEGF (pre) 0.270

Tenascin-C (pre) 0.270
